# Supplementary material for: Large freshwater phages with the potential to augment aerobic methane oxidation
Source: Nat Microbiol. 2020 Aug 24;5(12):1504–15. doi: 10.1038/s41564-020-0779-9 (PMC7674155; doi:10.1038/s41564-020-0779-9)
Supplement: Supplementary file 1 — Supplementary discussion, Figs. 1–25 and references. [file 41564_2020_779_MOESM1_ESM.pdf]

natureresearch

https://doi.org/10.1038/s41564-020-0779-9

# Supplementary information

# Large freshwater phages with the potential to augment aerobic methane oxidation

In the format provided by the authors and unedited

## Supplementary Discussion

### *SNPs analyses of pmoC-phages*

We found that the genome of BML_2 was remarkably well conserved both across and within samples. No SNPs were detected in the *pmoC* gene, indicating that this gene was highly conserved in the population. Only 10-37 base pair differences distinguish consensus sequences that were detected from each of the 13 samples. Between 14 and 160 SNPs were detected per sample. Segregating variants were found on average in 70% of the samples, indicating that many polymorphisms were consistent across samples. We categorized each SNP as non-synonymous (NS) or synonymous and found that the overall ratio of non-synonymous to synonymous SNPs was 0.75. Across all samples, there are 23 genes with at least one non-synonymous SNPs (Supplementary Fig. 15a), 14 of them are hypothetical proteins with no domain detected. Within the nine with predicted function are two DNA polymerases and one endonuclease encoded by syntenic genes (i.e., genes _45, _46 and _47; Supplementary Fig. 15a).

We found that variants that changed in frequency between the sampling year of 2016 and 2017 were about 2x as likely to be non-synonymous and that there were 14 non-synonymous variants that changed in frequencies between 2016 and 2017. These variants were found in three genes (Supplementary Fig. 15b). Two of these genes were unannotable and the third was an endonuclease with three amino acid variants that were present in most 2016 samples and absent from all but one 2017 sample. Overall, there was no average reduction or change in the average genetic diversity within the population between 2016 and 2017, indicating that any selective pressures present were not strong enough for selective sweeps of individual genotypes. Taken together, these results imply that the genes most quickly evolving in the phage population play largely unknown ecological roles.

### *Other genes relevant to host metabolism*

Some other genes reported in Cyanophage genomes [1] were detected in some phages reported here (Supplementary Fig. 22), including a heme oxygenase (*ho1*) and a *pcyX*-like phycocyanobilin:ferredoxin oxidoreductase that are involved in the production of phycobiliprotein pigment [2,3]. The *ho1* and *pcyX*-like genes are adjacent in TP6_1, BML_S_1 and CB_1 and one-gene-apart in CB_2 (Supplementary Fig. 23). One or more other genes for enzymes in porphyrin and chlorophyll metabolism (*bchE*, ALAS, *cobS*, *cobL*) were detected in some pmoC-phages and other related phages that lack *pmoC.* The transaldolase (*tal*) involved in the pentose phosphate pathway and reported in Cyanophages to enhance dNTP synthesis for phage replication [4], was encoded by five phages (4 are pmoC-phages). Additionally, genes for assimilatory sulfate reduction and a zeta toxin (a bactericide that inhibits cell wall biosynthesis) were present in some phages reported here.

## Supplementary Figures

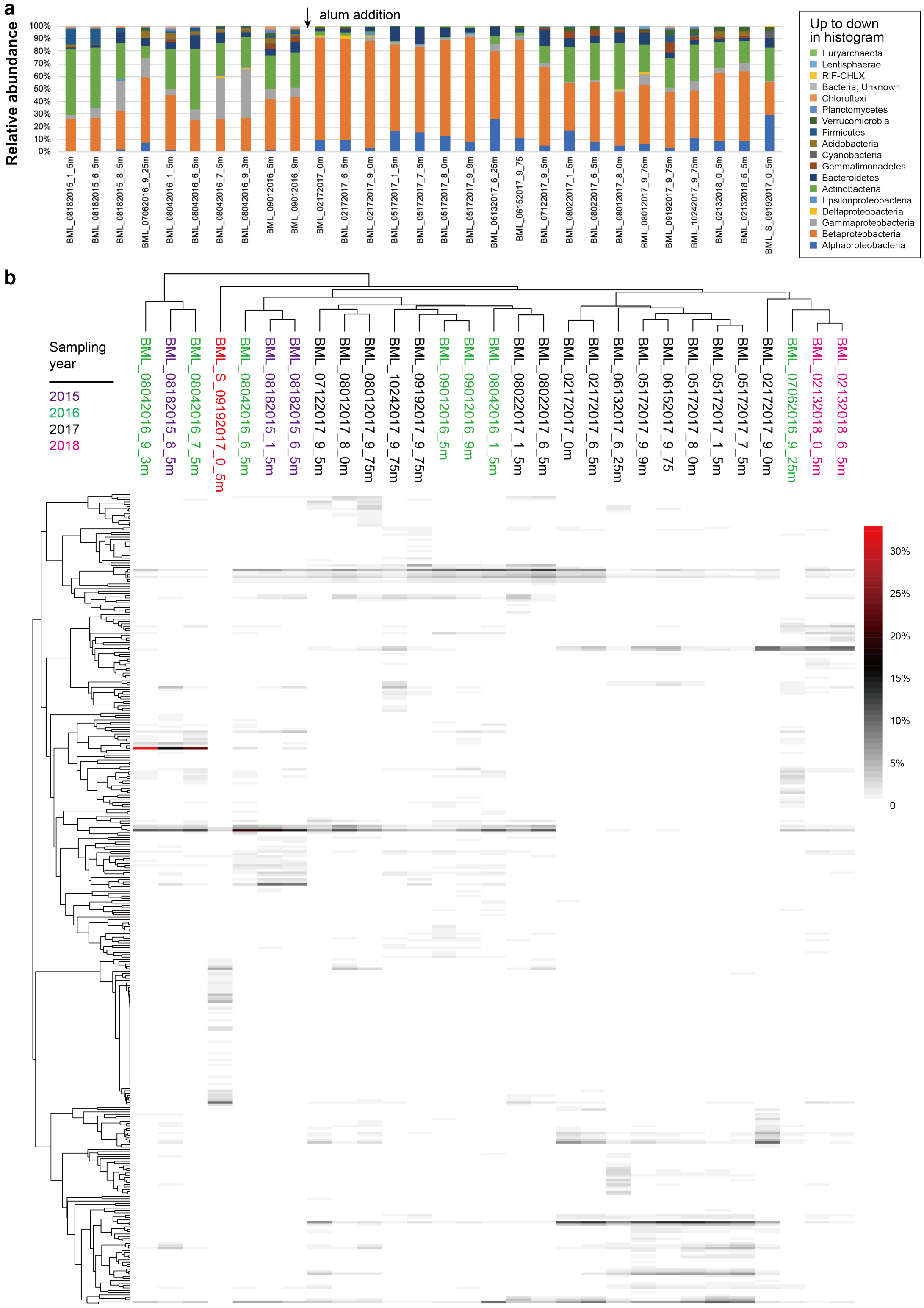


**Supplementary Fig. 1. Microbial community composition of BML and BML_S samples.** (a) The relative abundance of microbial phyla (or classes for Proteobacteria) in BML and BML_S samples. The analysis was performed based on ribosomal protein S3 (rpS3) genes (see methods in the main text for details). The addition of alum in 2016 was conducted to lower the available organic carbon concentrations in the water column. The communities were dominated by Actinobacteria, Alphaproteobacteria, and Betaproteobacteria. (b) The clustering analyses of BML and BML_S samples based on the relative abundance of microbial species/strain (determined based on rpS3) detected in the samples. Note that the BML_S microbial community is very different from the BML communities. The sampling year is indicated by different colors. The clustering was performed using the R package of “pheatmap” [5] with the “correlation” clustering algorithm and “average” method.

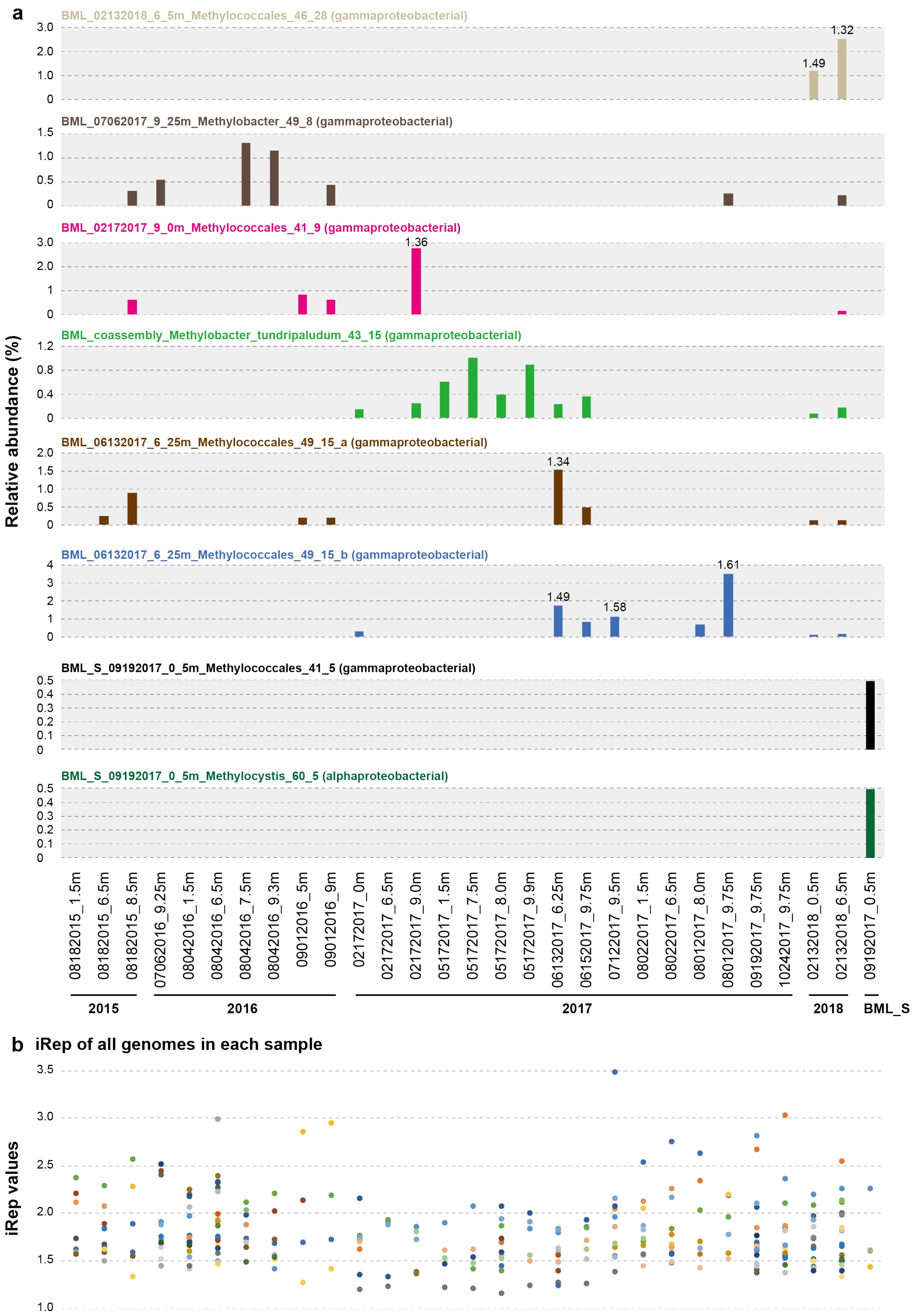


**Supplementary Fig. 2. The growth rate of microorganisms detected in BML and BML_S samples.** (a) The growth rate (iRep values) and relative abundance of alphaproteobacterial and gammaproteobacterial methanotrophs. The taxonomic information for methanotrophs is shown in the brackets, the iRep values are shown above the bars indicating relative abundance. (b) The growth rate (iRep values) of all microorganisms. Growth rates via iRep analyses were only estimated using genomes with at least a 5X coverage.

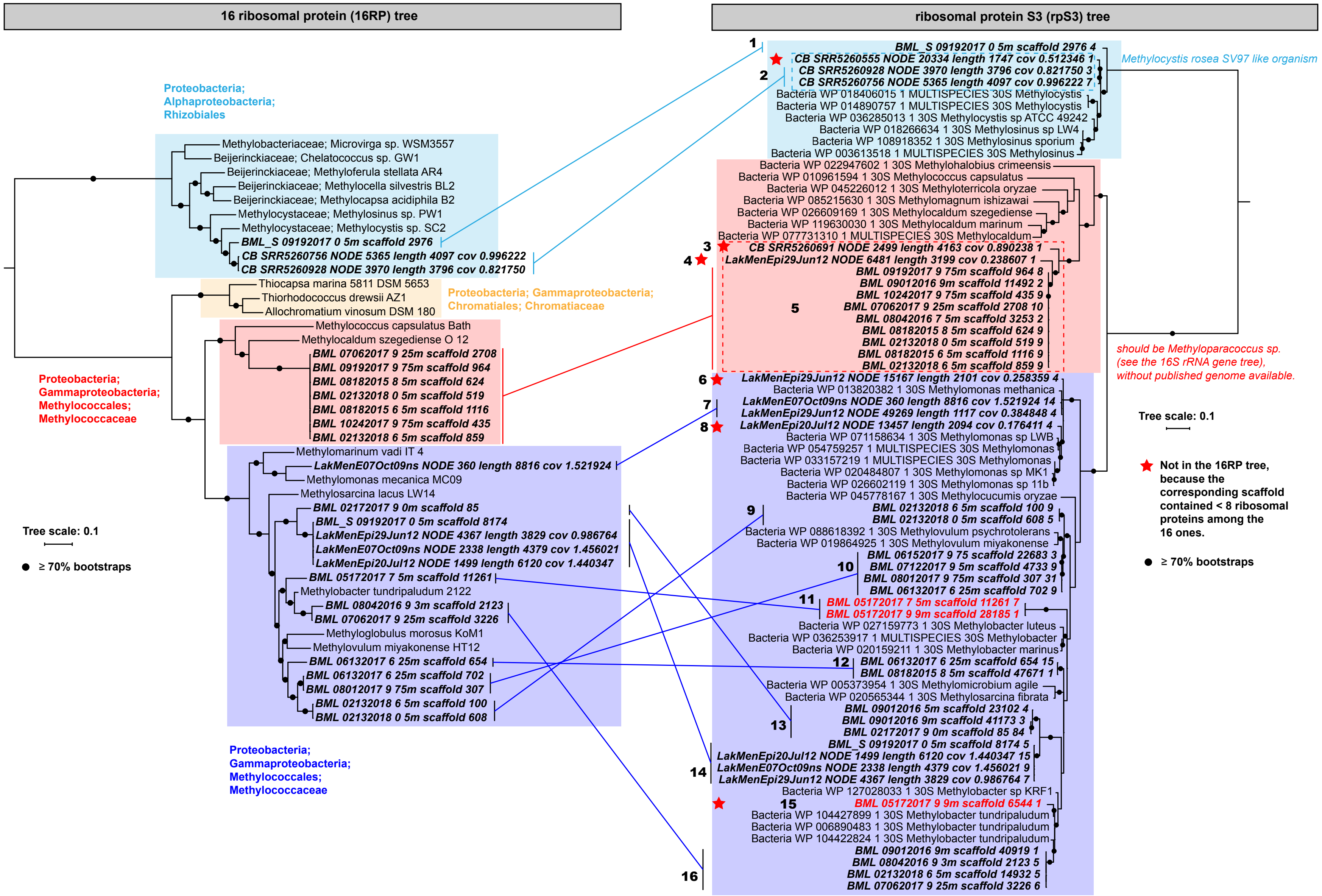


**Supplementary Fig. 3**. Phylogenetic analyses of bacterial methanotrophs based on (a) concatenated sequences of 16 ribosomal proteins (16RPs) and (b) the ribosomal protein S3 (rpS3). The scaffolds in both trees are linked by lines and marked with a number for reference to the 16S rRNA tree (see **Supplementary Fig. 4**). The scaffolds with 7 or fewer of the 16RPs were not included, as indicated by red stars in the rpS3 tree. The major lineages are highlighted in colored backgrounds. References were selected based on the rpS3 BLASTp search (top 5 hits for each). For a summary based on all three phylogenetic analyses: alphaproteobacterial methanotrophs were detected in CB and BML_S samples, gammaproteobacterial methanotrophs were detected in BML, BML_S, CB and LM samples (which are in bold).Partial rpS3 protein sequences are in red. Note that the proteins and 16S rRNA genes were predicted from scaffolds with a minimum length of 1 kbp.

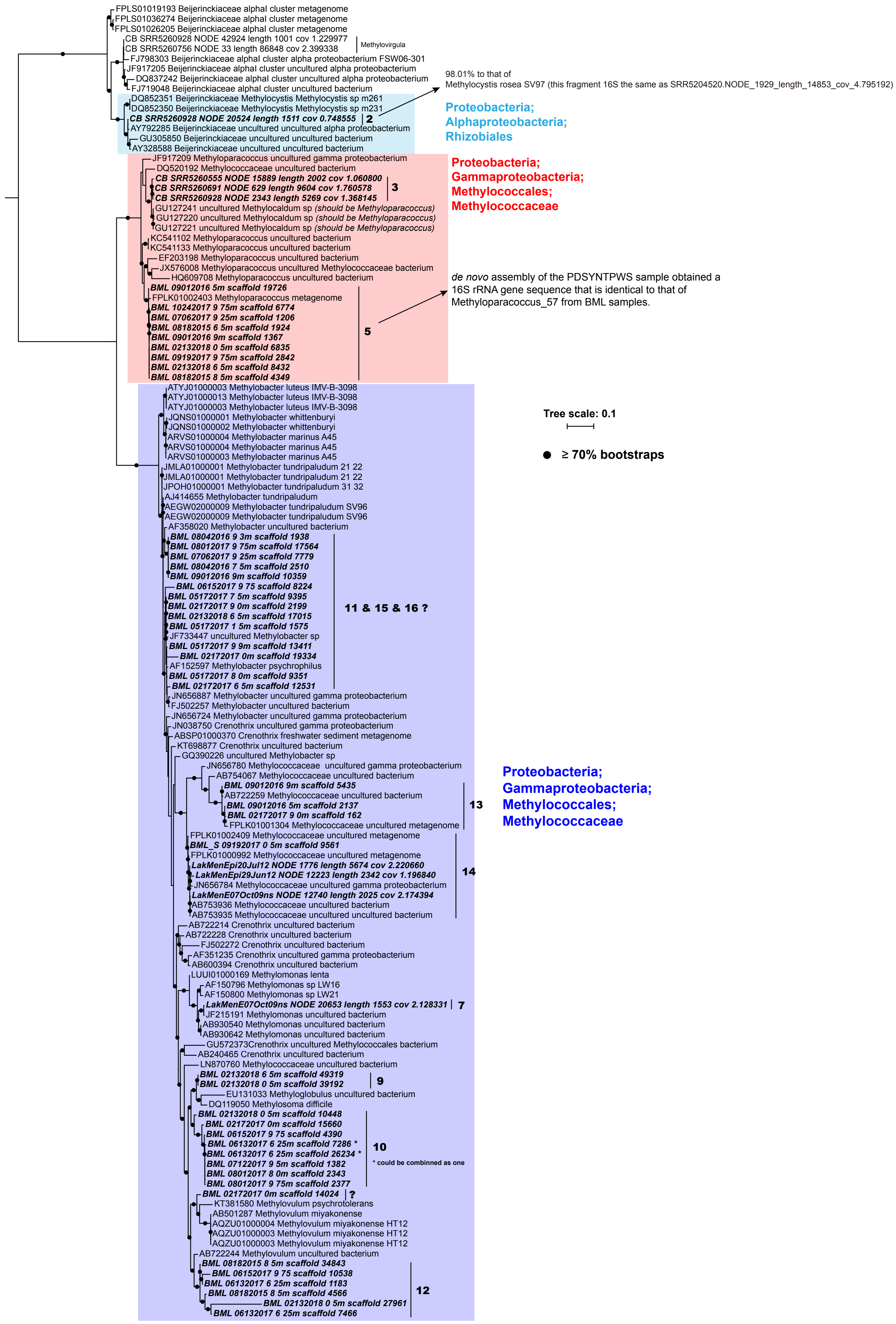


**Supplementary Fig. 4**. Phylogenetic analyses of bacterial methanotrophs based on the 16S rRNA gene sequences. Ones without a match in 16RP or rpS3 tree are indicated a question mark.

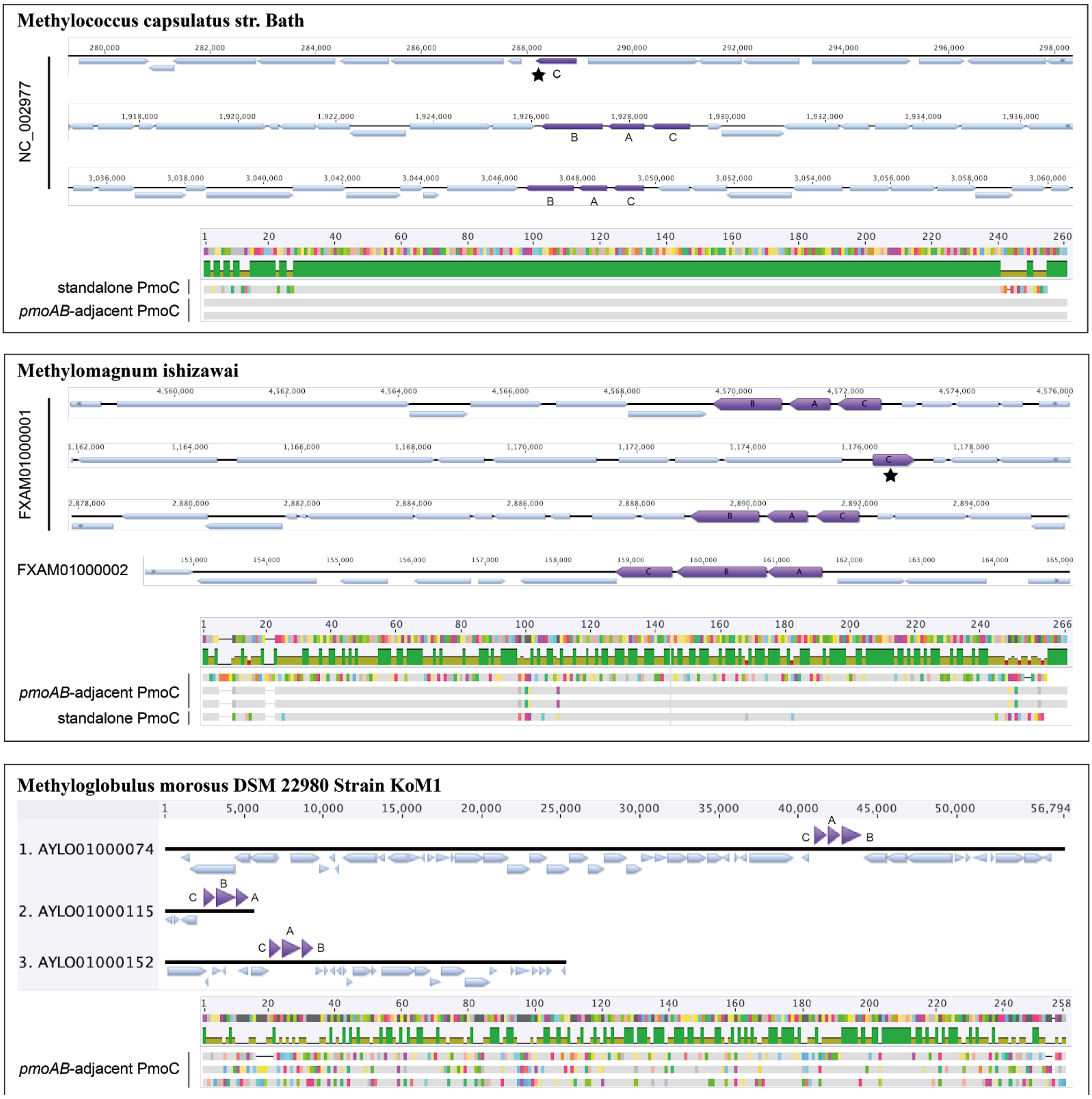


**Supplementary Fig. 5. The pMMO subunits detected in published bacterial methanotroph genomes.** The order of three subunits may vary from operon to operon (*pmoACB* or *pmoABC*). The protein sequence alignment of *pmoAB*-adjacent and standalone PmoC is shown. It is interesting that the PmoC from the same genome are divergent from each other, or the standalone PmoC can be very similar to the ones within an operon. For analyses, the Genbank files of the genomes were downloaded from NCBI RefSeq, and the figures were constructed in Geneious with the pMMO subunits highlighted based on Genbank annotations. The standalone of *pmoC* genes are indicated by black stars.

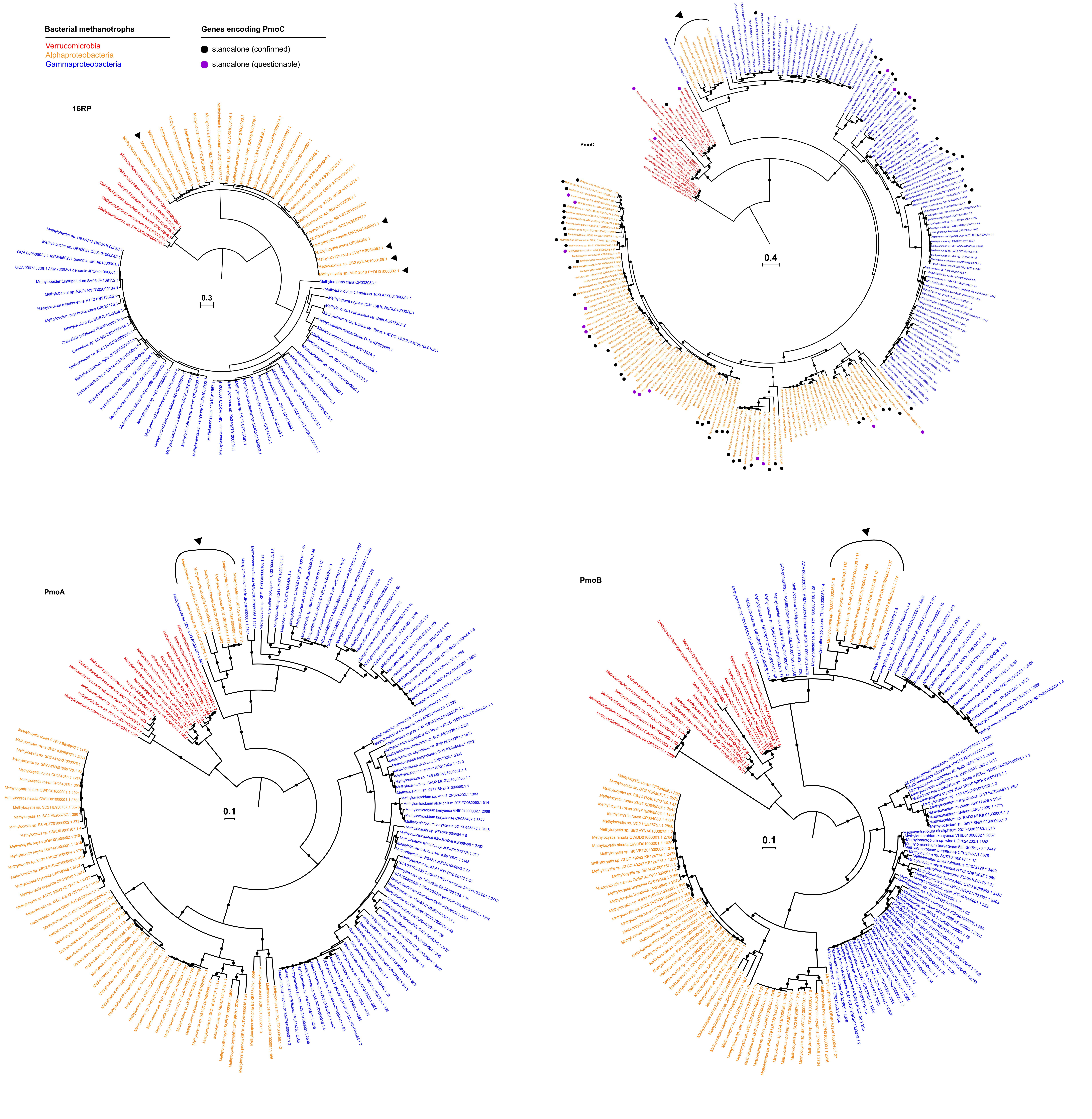


**Supplementary Fig. 6.** Phylogenetic analyses of published bacterial methanotrophs based on concatenated sequences of 16 ribosomal proteins and pMMO subunits (PmoC, PmoA, and PmoB). Only those genomes with a scaffold containing eight or more of the 16 ribosomal proteins are included for the 16RP-based phylogenetic analyses. The alphaproteobacterial methanotrophs with one copy of pMMOs phylogenetically clustered with some gammaproteobacterial methanotrophs, as indicated by black triangles. Some bacterial methanotrophs genomes encode standalone pmoC (without pmoA/pmoB nearby), as confirmed via analysis of genetic context are indicated by black circles, and pmoC genes detected at the end of scaffolds (questionable "standalone") are indicated by purple circles. A dot on the tree indicates bootstrap value ≥ 70.

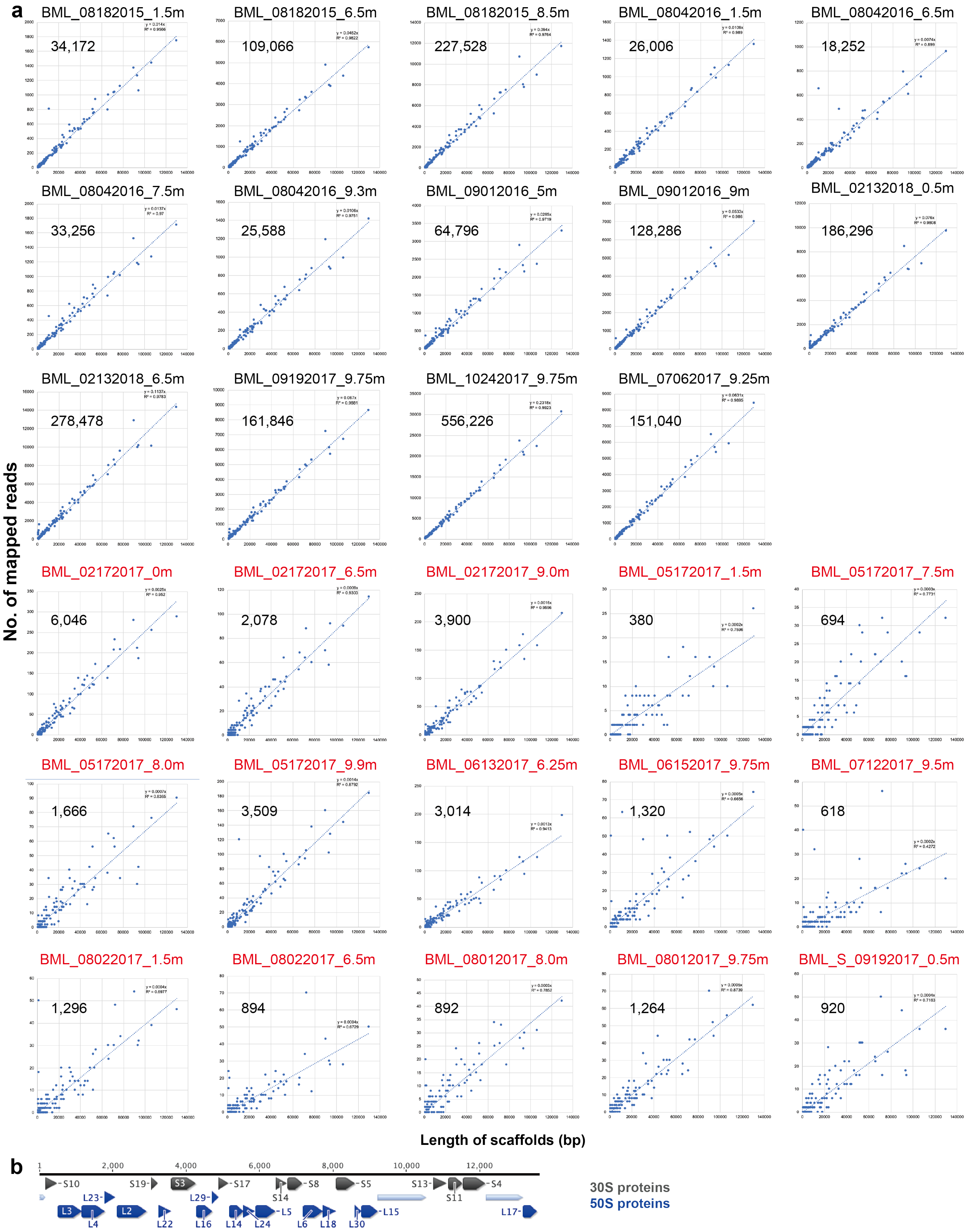


**Supplementary Fig. 7. The detection of Methyloparacoccus_57 at low abundance in some BML samples.** For each sample, the number of mapped reads to each scaffold was plotted as the function of the length of the corresponding scaffold. The total number of reads mapped to Methyloparacoccus_57 in each sample is shown. The sample names shown in black contained Methyloparacoccus_57, based on detection of assembled scaffolds and reads mapping, whereas those ones shown in red had Methyloparacoccus_57 detected only by reads mapping. See “The confirmation of Methyloparacoccus_57 with low abundance in some BML samples” in the Methods section for details.

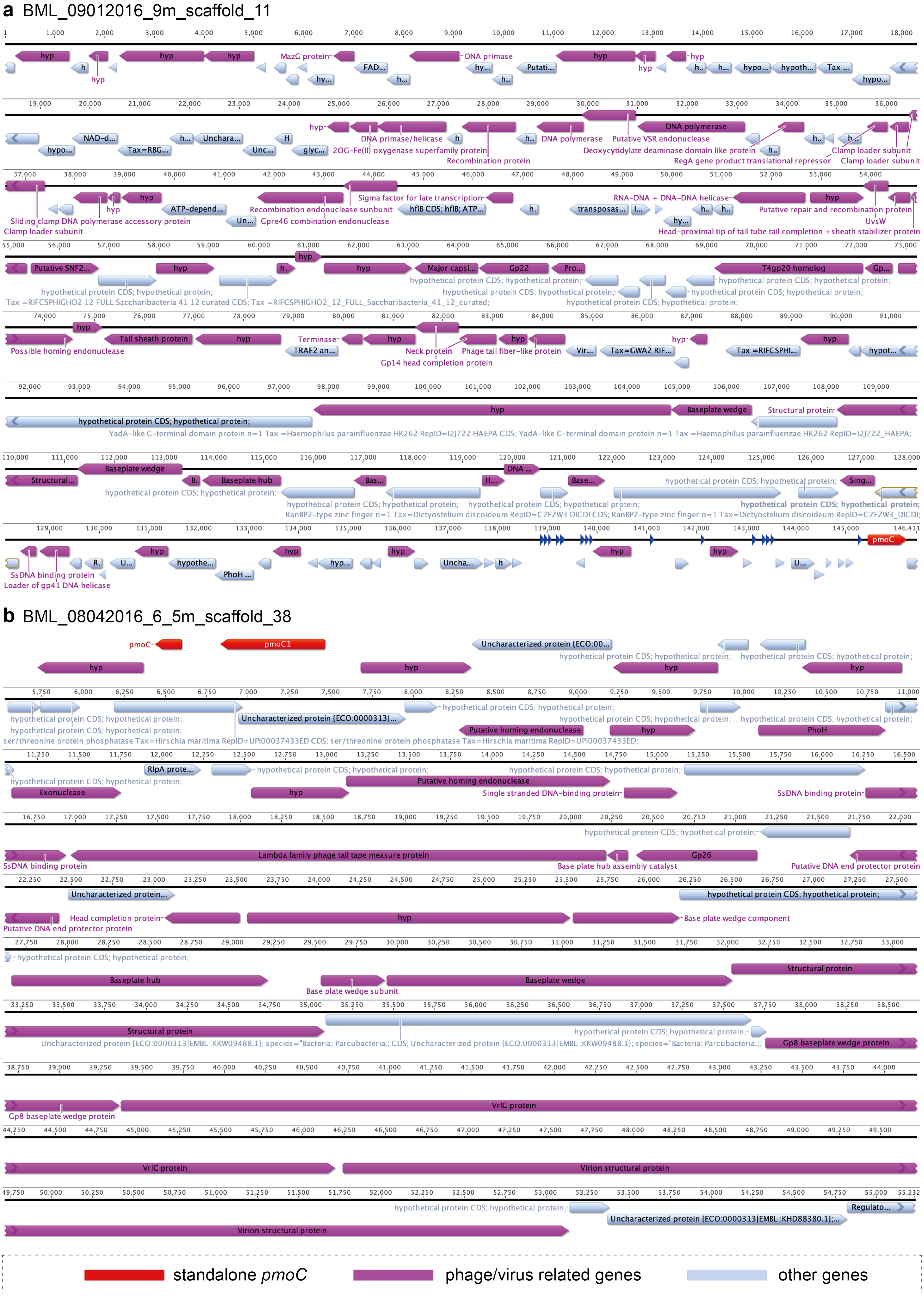


**Supplementary Fig. 8. Examples of *pmoC* gene detected on scaffolds with phage genes.** The phage/virus related genes were identified by the search against the KEGG, UniRef and UniProt databases (see Methods in the main text for details).

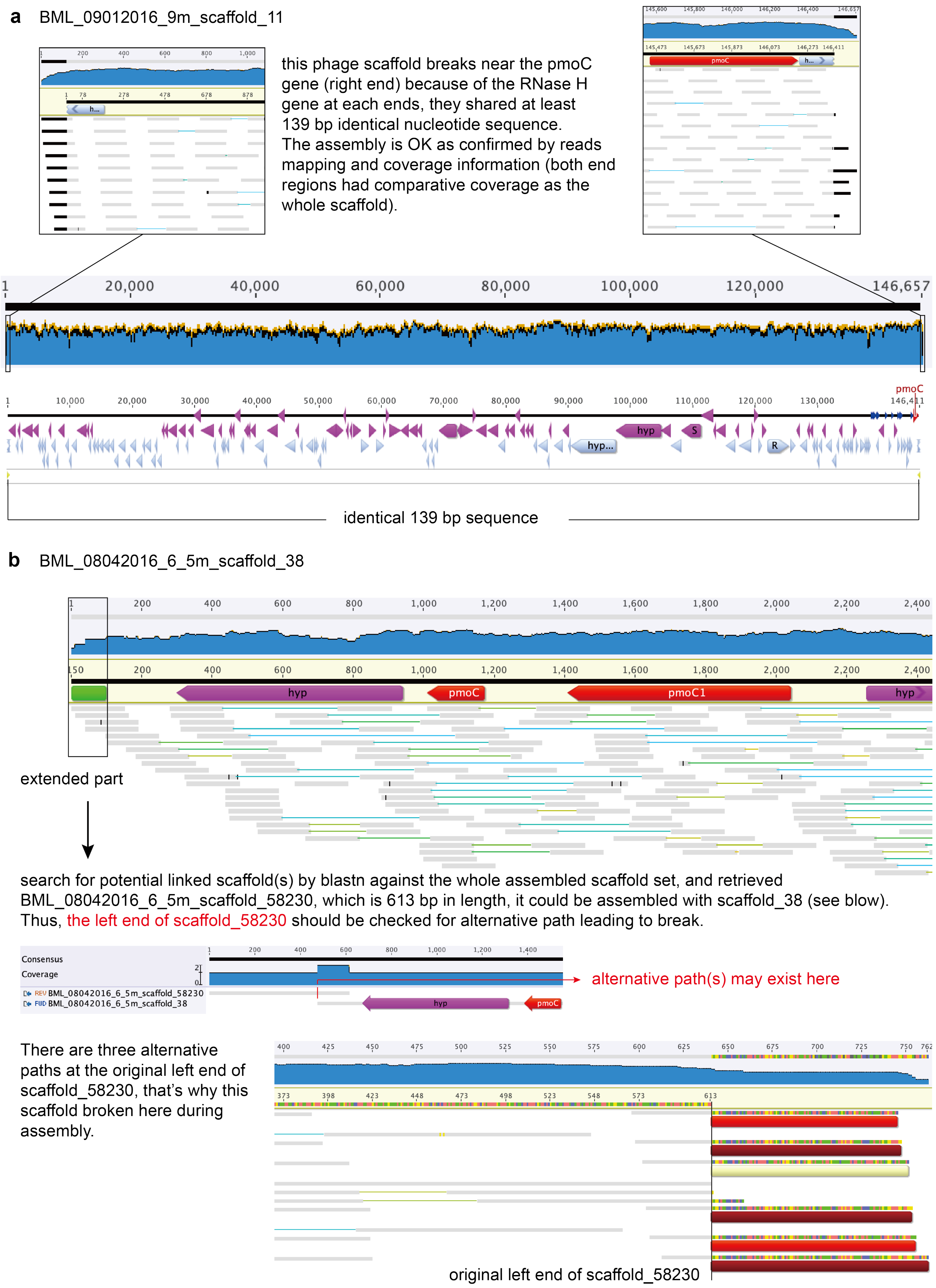


**Supplementary Fig. 9. Examples showing the reasons why phage scaffolds broke at or near the *pmoC* gene.** In detail, the example (a) is due to the repeat sequences at the two ends of the scaffolds, (b) is due to the existence of alternative paths.

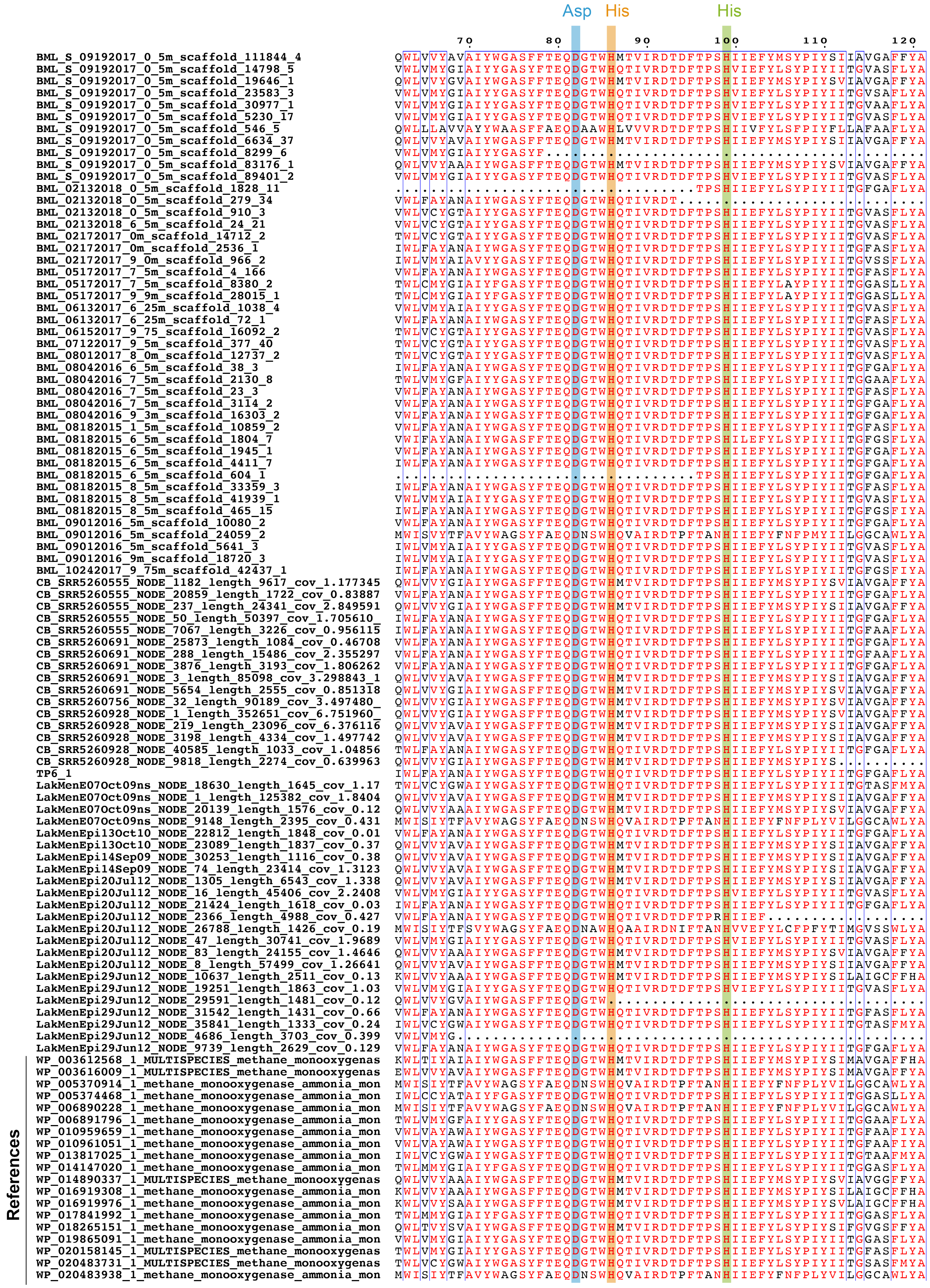


**Supplementary Fig. 10. Alignment of bacterial and phage-associated PmoC sequences detected in BML, BML_S, LM and CB samples.** The residues involved in $O_2$ binding and methane oxidation are highlighted.

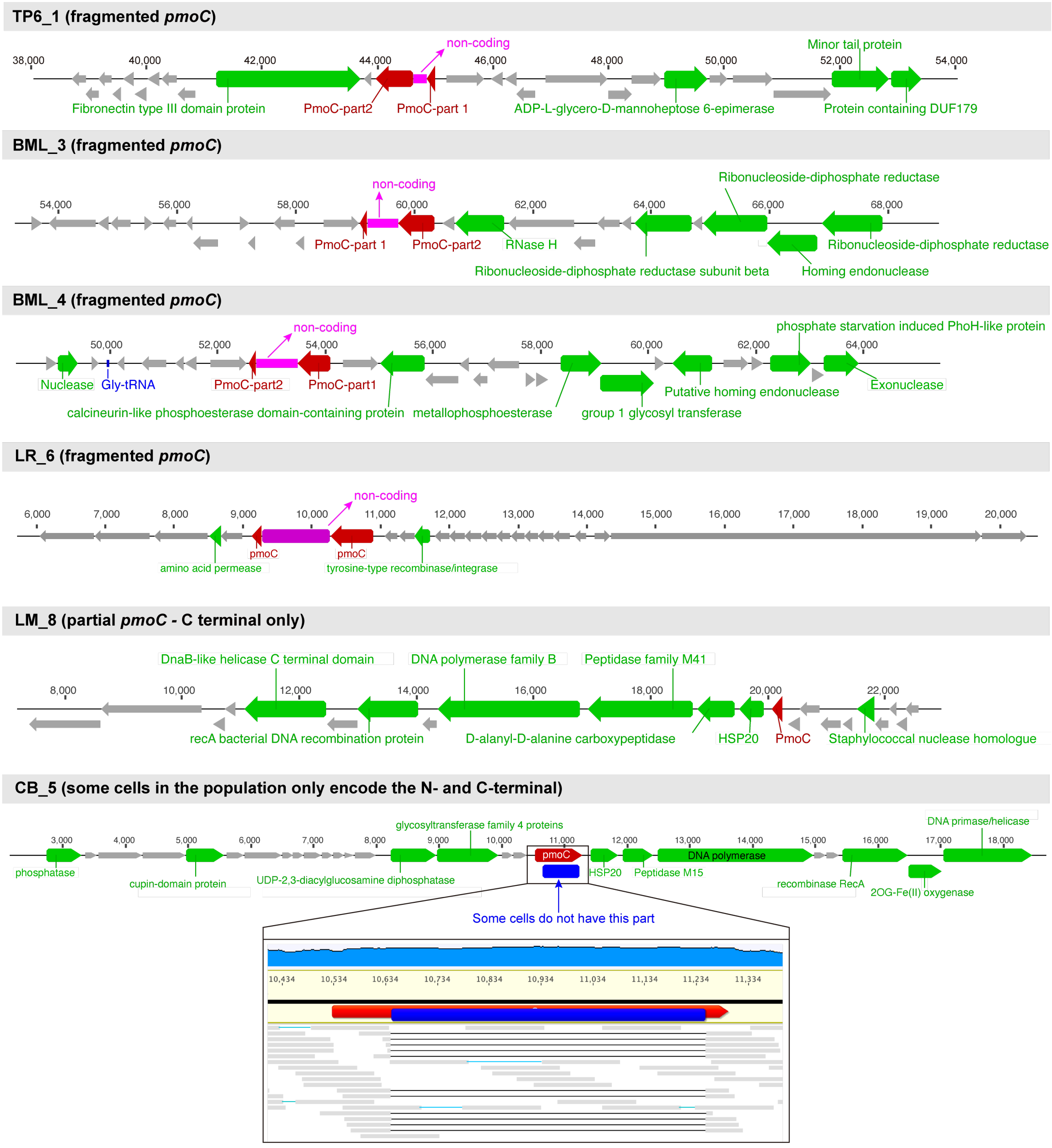


**Supplementary Fig. 11. Genetic context of fragmented and partial *pmoC* genes in pmoC-phages.** Fragmented or partial phage-associated PmoC were detected in pmoC-phages with predicted hosts of an alphaproteobacterial methanotroph (i.e., LM_8, CB_5) and gammaproteobacterial methanotrophs (i.e., TP6_1, BML_3 and BML_4). The protein-coding genes with functional annotations are shown in green, the ones without functional annotation in grey, tRNA in blue. Some CB_5 cells contained only part of the *pmoC* gene, which is shown in detail (black lines connect aligned portions of reads).

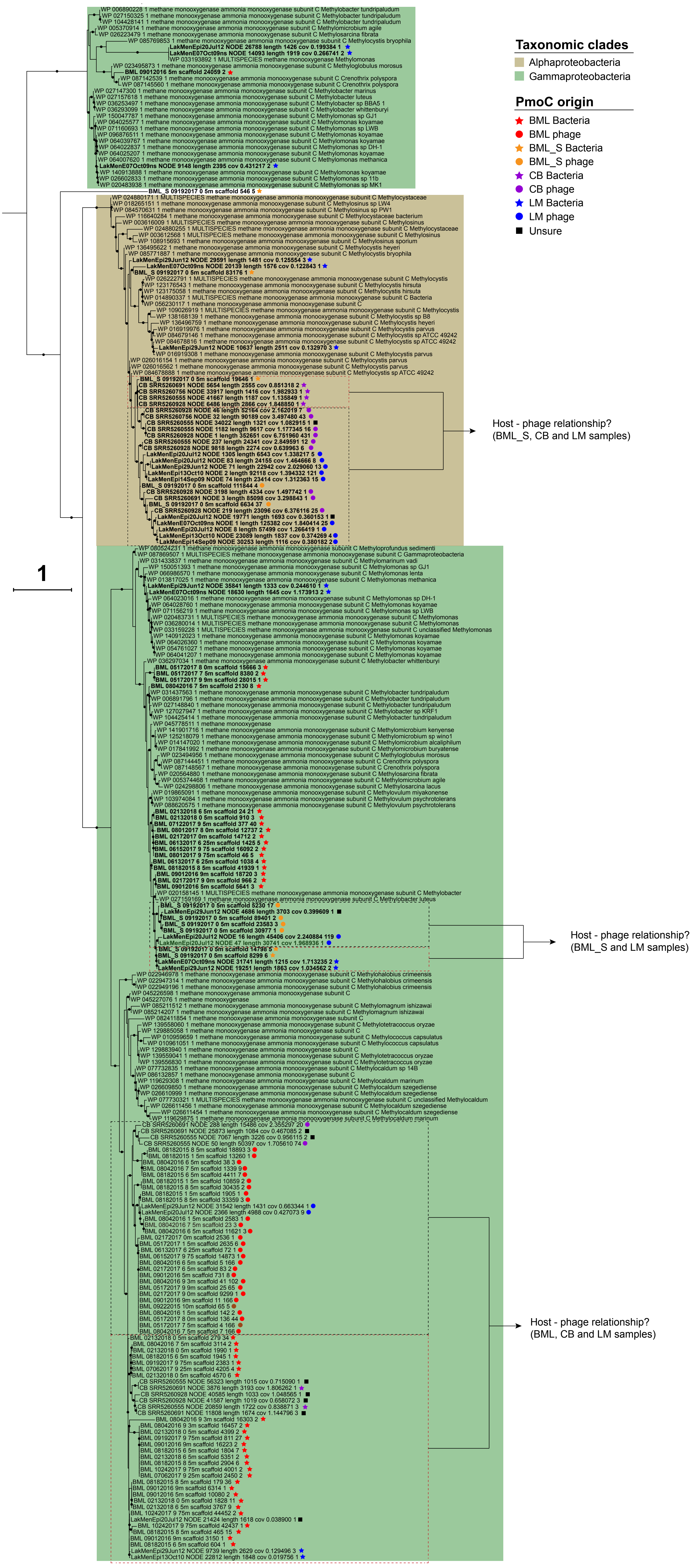


**Supplementary Fig. 12**. The full phylogenetic tree of bacterial and phage-associated PmoC detected in BML, BML_S, LM, CB and TBL samples. PmoC from published bacterial methanotrophs with genomes available are included for reference, see Supplementary Tables 3, 4 and 6 for details. Please note that only a subset of metagenomic datasets from LM and CB have been re-analyzed and their PmoC were included here. All those reported in this study are in bold.

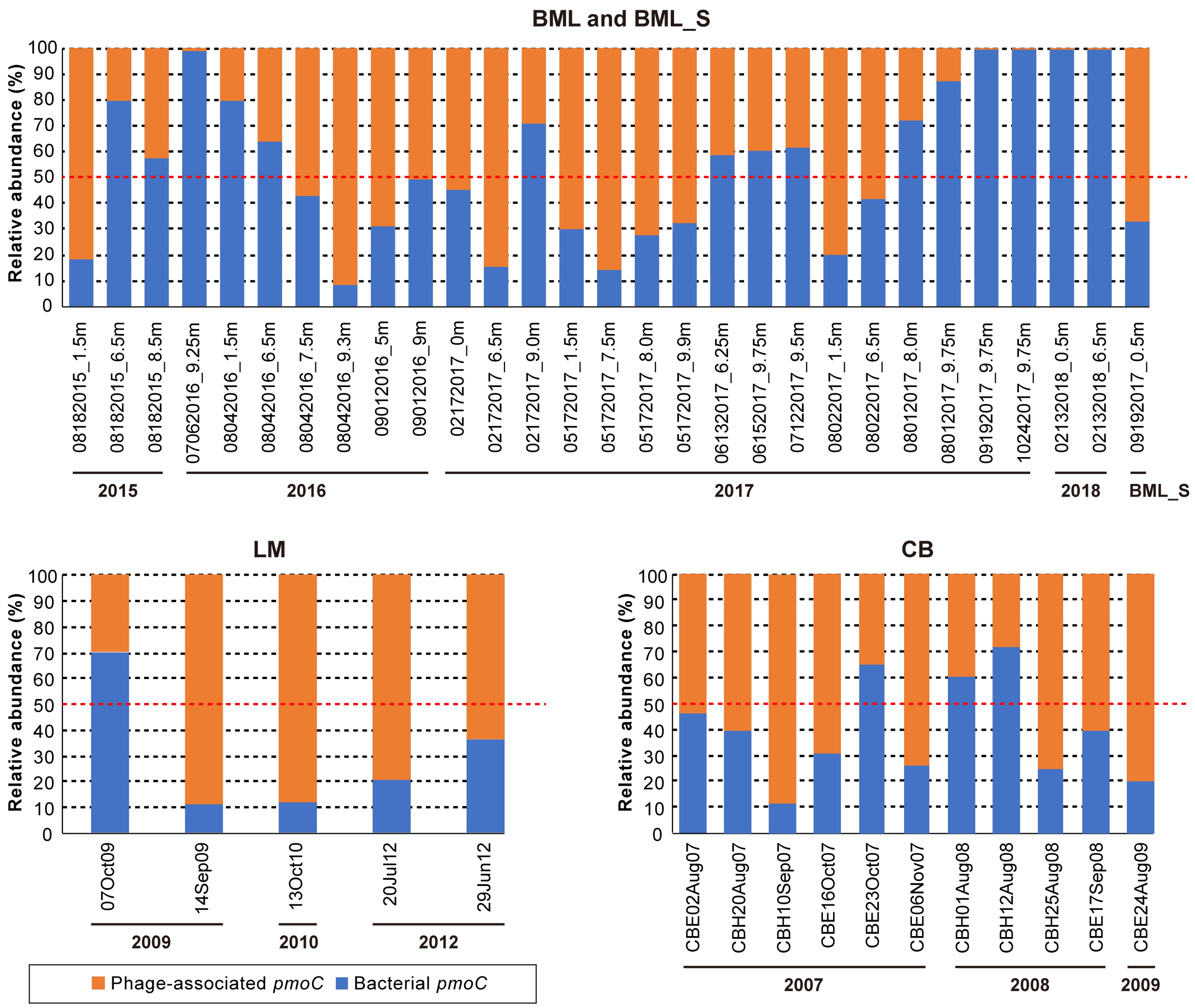


**Supplementary Fig. 13.** The cumulative relative abundances of bacterial and phage-associated *pmoC* genes in samples from BML, BML_S, LM, and CB. Please note that only some of the metagenomic datasets from LM and CB were re-analyzed, and data for those is shown here. The quality reads from each sample were mapped to the representative scaffolds with a detected bacterial or phage-associated *pmoC* gene, and the sequencing coverage was calculated for each scaffold, the cumulative coverage of all bacterial pmoC scaffolds was summed as $i$, and that of phage-associated pmoC scaffolds summed as $j$, the cumulative relative abundance of bacterial *pmoC* was calculated as $i/(i + j)$ X 100%, and that of phage-associated *pmoC* was calculated as $j/(i + j)$ X 100% (or 1 - $i/(i + j)$ X 100%). The red dash line indicates an equal relative abundance of bacterial and phage-associated *pmoC*.

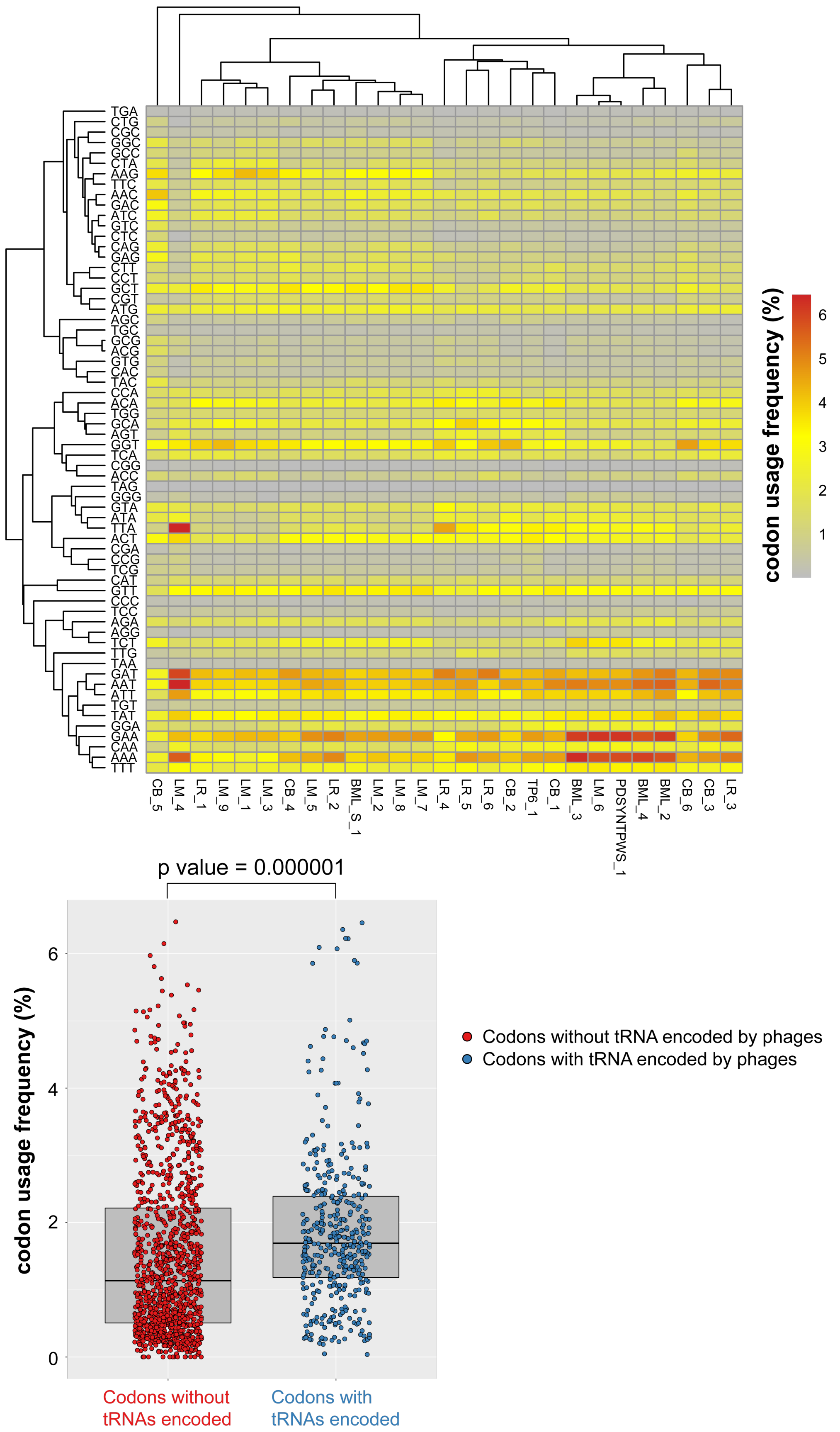


**Supplementary Fig. 14. The codon usage frequency of pmoC-phages and their relatives.** Upper panel: Clustering analyses of phages based on codon usage frequency. Bottom panel: Comparison of usage frequency of codons without tRNA encoded and with tRNA encoded in the phages. The ones with tRNA encoded by phages showed an overall higher usage frequency at 95% confidence level (two-sided unpaired student's t-test; P value = 0.000001). Each point represents the usage frequency of a given codon in a given phage. Box plots enclose 1st to 3rd quartiles of data values, with a black line at the median value.

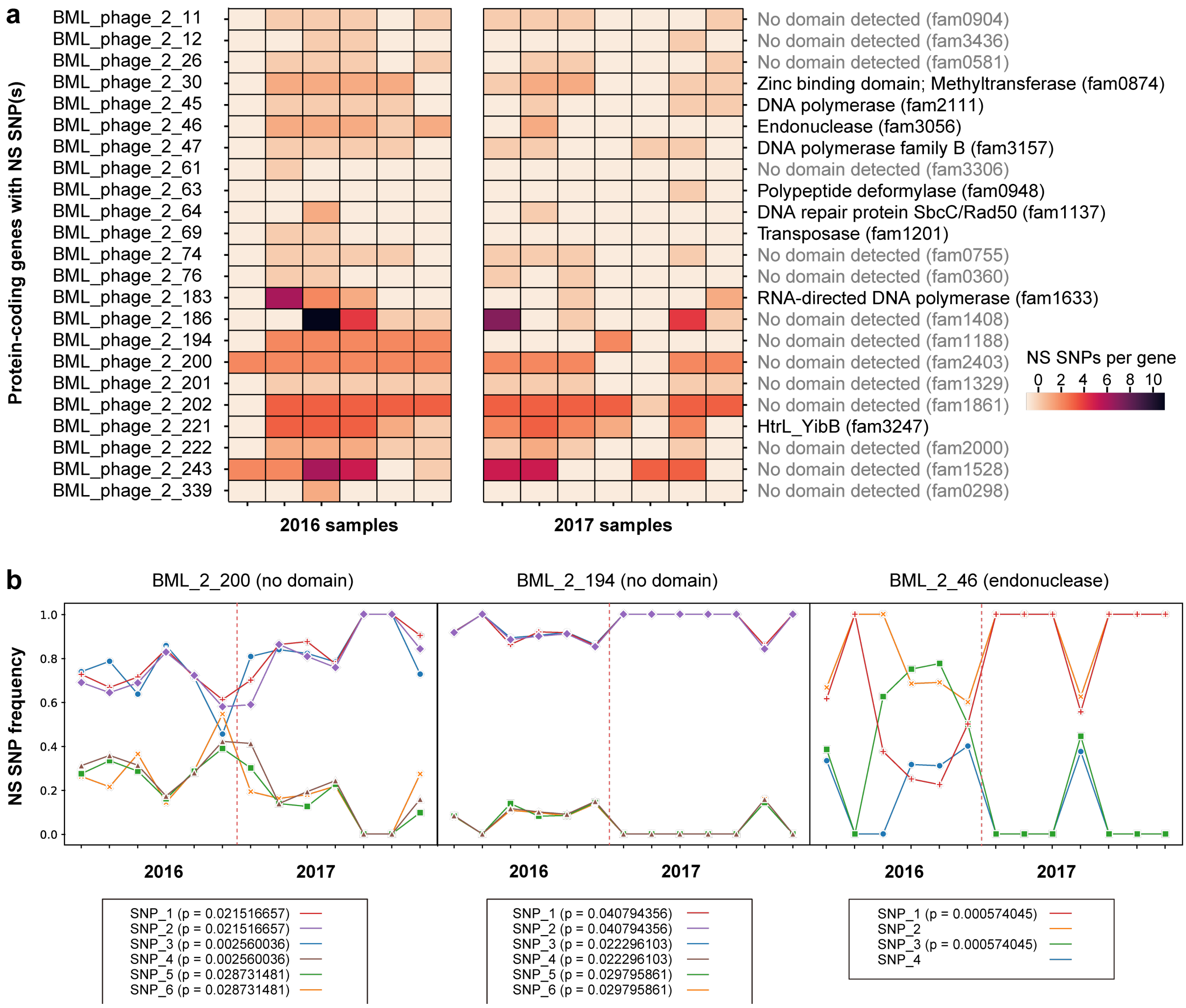


**Supplementary Fig. 15. Observed SNP frequencies of pmoC-phage BML_2.** (a) The number of SNPs detected in protein-coding genes and their functional annotation. Only protein-coding genes with non-synonymous (NS) SNPs are shown and listed in the order of the genes on the chromosome. The annotations of protein-coding genes and their corresponding protein families are listed on the right. (b) Frequencies of NS SNPs in genes with SNPs that changed significantly (two-sided z-test of proportions; $p < 0.05$) between 2016 and 2017 samples. Each allele and its corresponding alternative allele are represented by a line, and SNPs are grouped by the origin of the genes. The p values are shown in the figure legend when < 0.05.

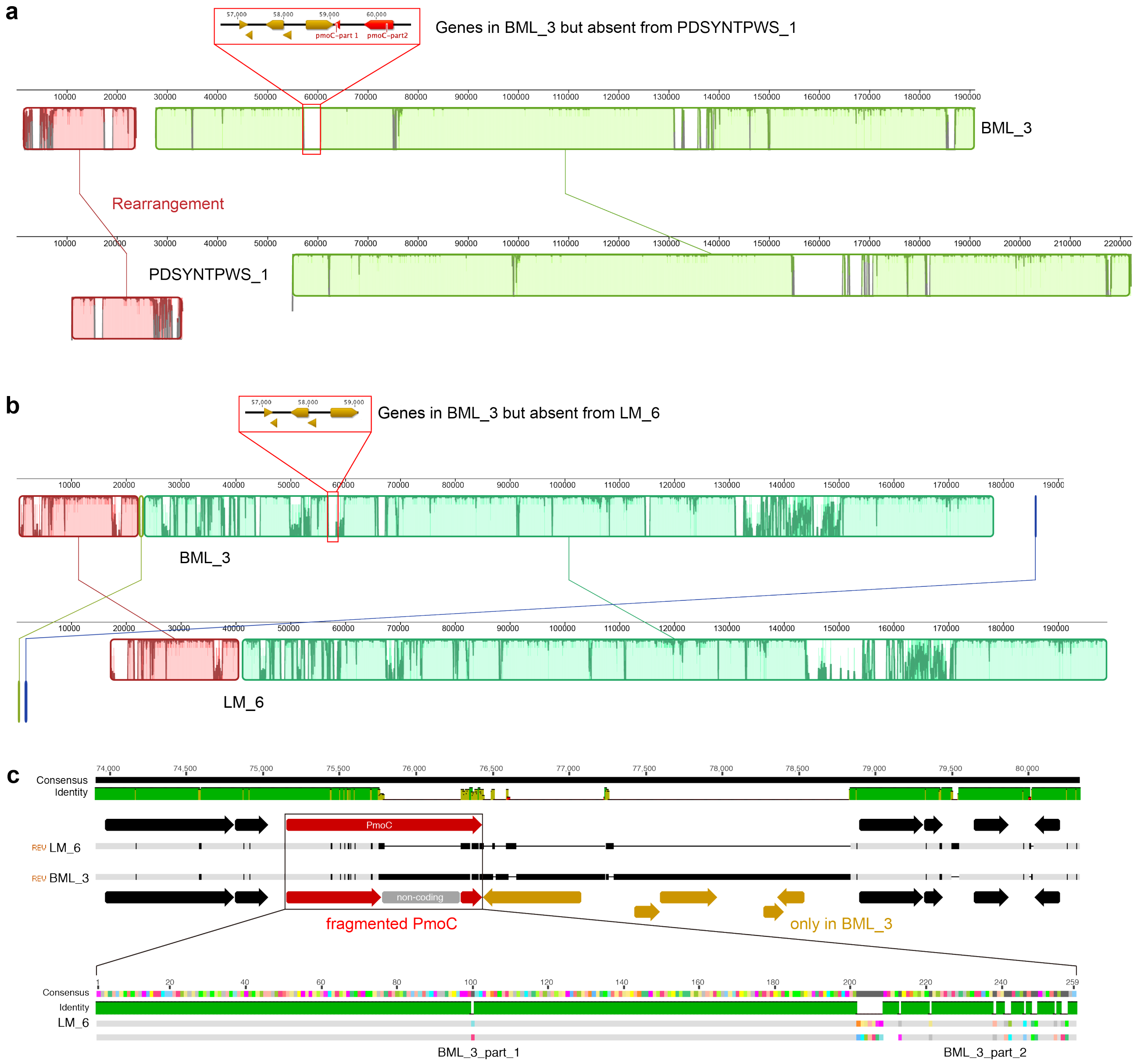


**Supplementary Fig. 16. Whole-genome alignment of BML_3, PDSYNTPWS_1 and LM_6.** (a) Mauve genome alignment of BML_3 and PDSYNTPWS_1. The pmoC gene and five syntenic genes (all hypothetical proteins) that are present in BML_3 while absent from PDSYNTPWS_1 are shown in detail at the top. (b) Mauve genome alignment of BML_3 and LM_6. The five syntenic genes absent from LM_6 are shown at the top. (c) Genome alignment of the pmoC regions of BML_3 and LM_6. Five protein-coding genes near the fragmented PmoC are only present in BML_3. The alignment of PmoC sequences shows the breakpoint of the PmoC in BML_3. Note that the fragment containing the N-terminus of PmoC of BML_3 is with only one amino acid divergence with that of LM_6, while their C-terminus sequences are very divergent.

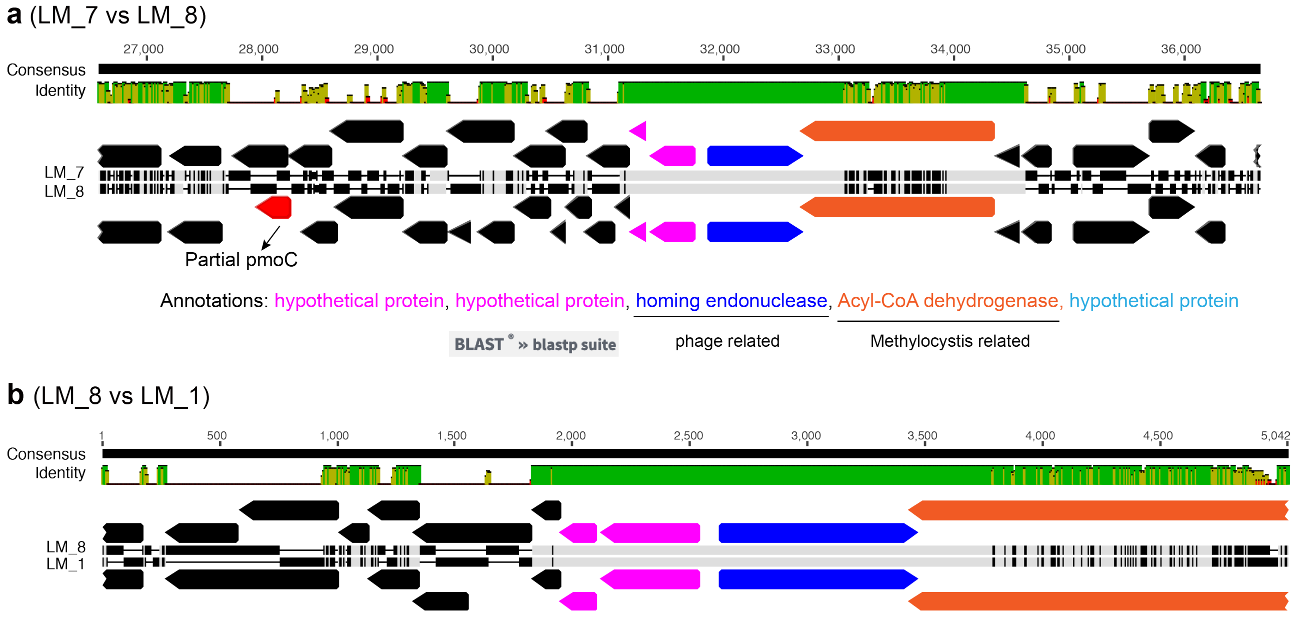


**Supplementary Fig. 17. The shared region among LM_1, LM_7 and LM_8.** Manual curation was performed to confirm that these phage genomes share this region. The NCBI BLASTp information is shown for the two protein-coding genes that could be annotated.

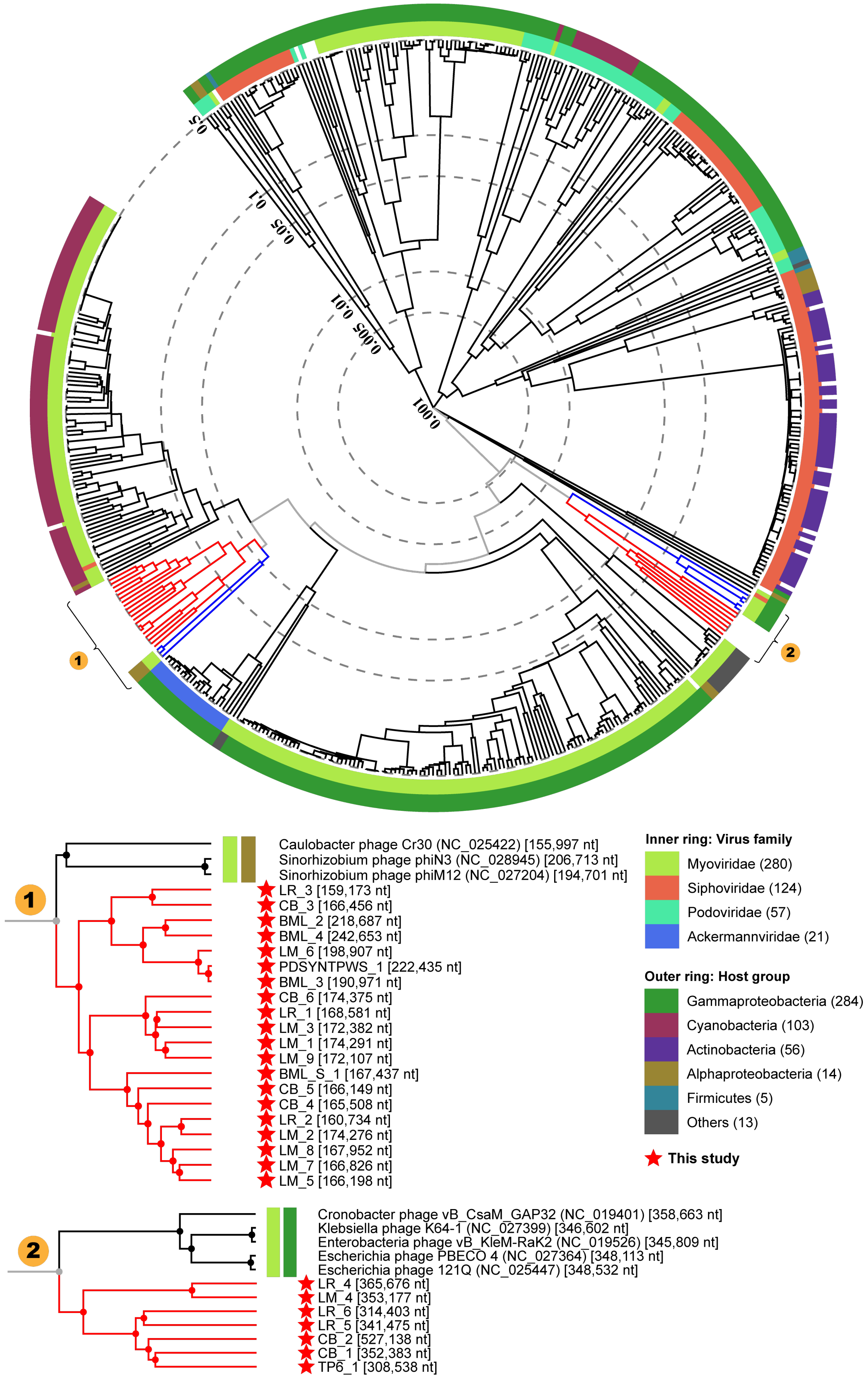


**Supplementary Fig. 18. The selection of reference virus genomes based on Viptree analyses.** The analysis was performed by uploading the curated phage genomes reconstructed in this study via the viptree online analysis tool (https://www.genome.jp/viptree/), which generated the circular proteomic tree. Based on this, the reference virus/phage genomes with protein profiles similar to those of phages reported in this study are shown in detail at the bottom of the Figure. These reference genomes were used for protein family and phylogenetic analyses (see main text).

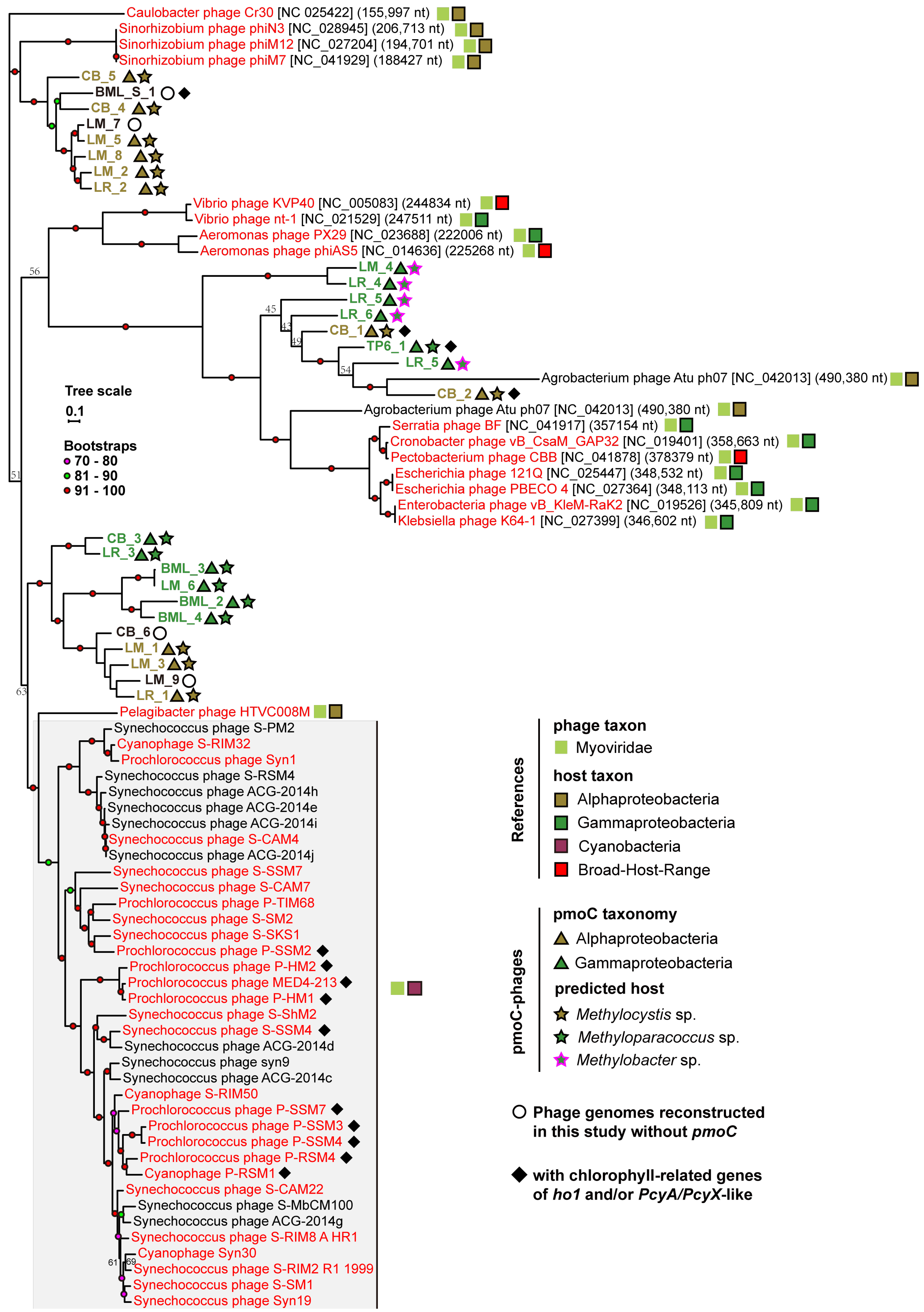


**Supplementary Fig. 19. Phylogenetic analyses of phages with genomes reported in this study based on DNA polymerases.** The taxa of reference phages and their hosts are shown with information from the NCBI virus-host database [6]. The bootstraps ≤ 70 are shown in numbers.

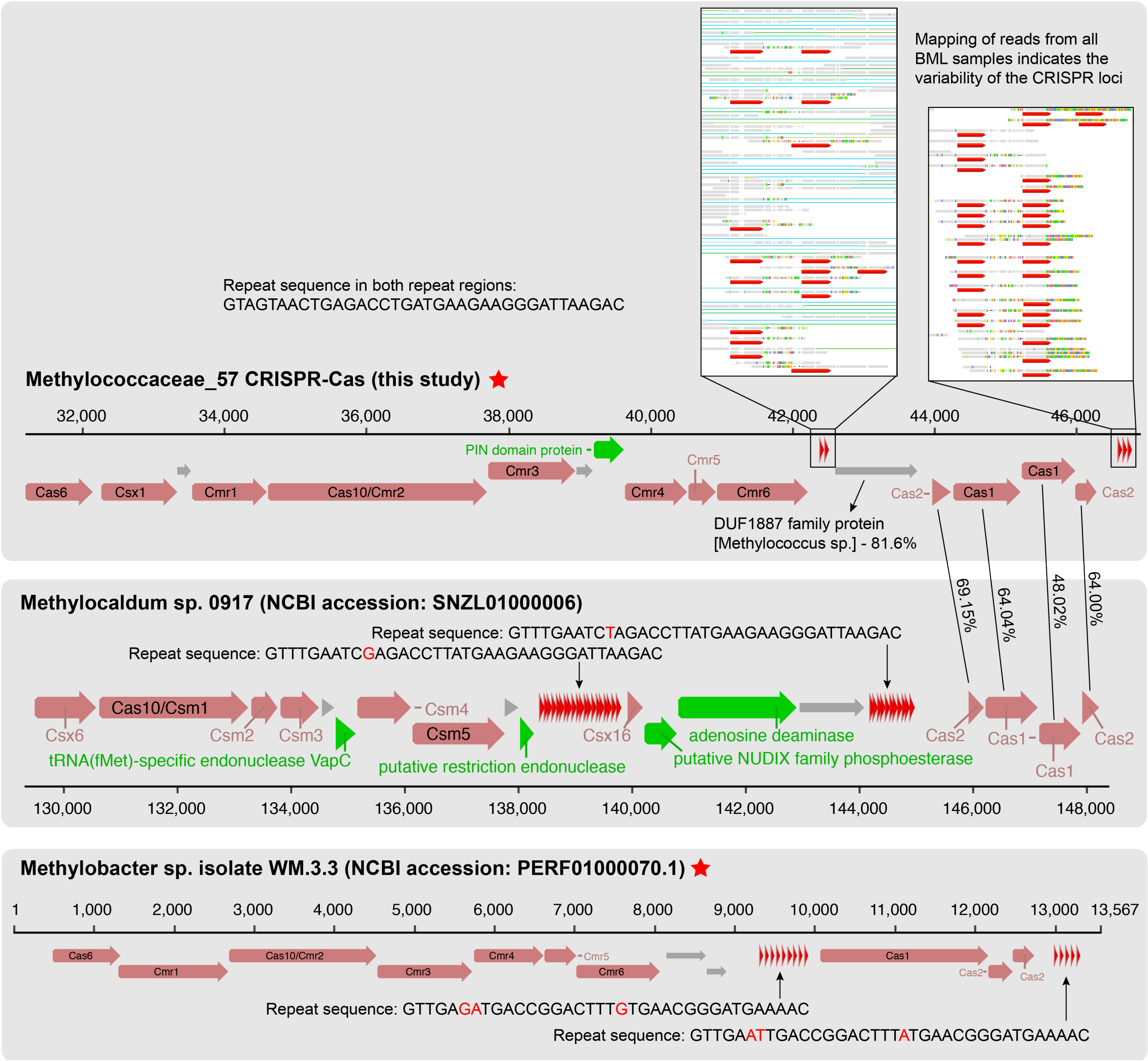


**Supplementary Fig. 20. CRISPR-Cas analyses of bacterial methanotrophs reported in this study and that already published.** Methyloparacoccus_57 (top panel) contains two CRISPR loci with the same direct repeat sequence. The Cas1 and Cas2 protein sequences are most similar to those from Methylocaldum sp. 0917 (middle panel), which also has two repeat regions and the repeat sequences are only one base different (shown in red). The mapping of reads from all BML samples to the CRISPR scaffolds indicates divergences in spacer sequences, and we found one of the spacers matches the genomic sequence of pmoC-phage BML_4. Interestingly, one spacer from the published *Methylobacteria* sp. isolate WN.3.3 (bottom panel) also targets pmoC-phage BML_4. The CRISPR-Cas system of *Methylobacteria* sp. isolate WN.3.3 is also similar to that of Methyloparacoccus_57 as it shares the same type of other Cas proteins, except Cas1 and Cas2.

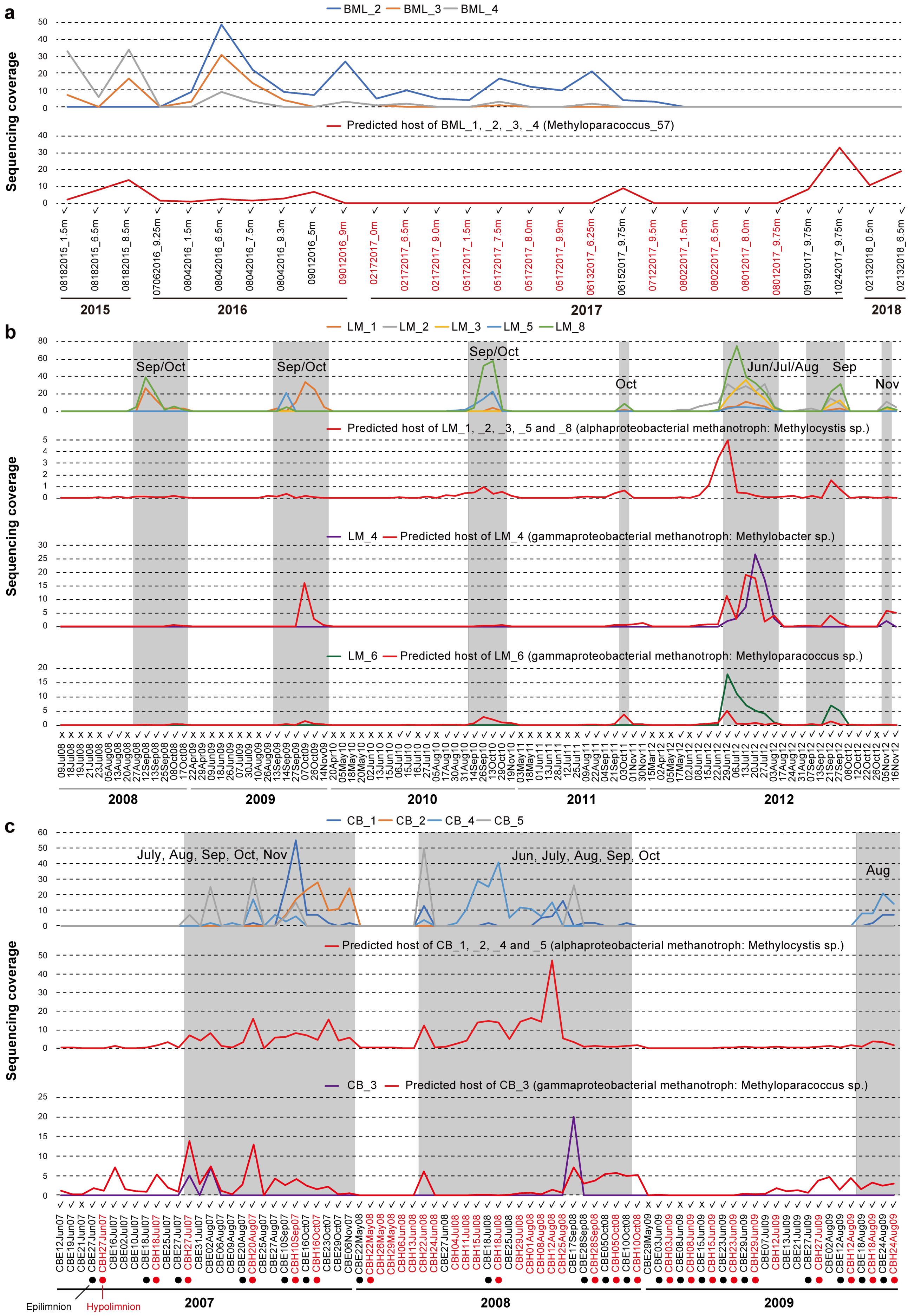


See next page for legend

**Supplementary Fig. 21. The sequencing coverage profiles of genomes of pmoC-phages and their predicted hosts in (a) BML, (b) Lake Mendota and (c) Crystal Bog samples.** The published pmoC-phage of TP6_1 sampled in August 2011 from 0-10 cm of an oil sands lake [7] is predicted to have the same host as other BML pmoC-phages. The names of samples detected with very low abundances of Methyloparacoccus_57 are shown in red (see Supplementary Fig. 7 and Methods in the main text for details). Grey shading indicates times when both pmoC-phages and the predicted host are relatively abundant. When one bacterium was predicted as the host of multiple pmoC-phages, their profiles are shown in separate panels. The epilimnion and hypolimnion samples collected on the same day from Crystal Bog are paired and indicated by solid black and red circles, respectively. Only epilimnion samples were collected from Lake Mendota. A “✓” indicated the detection of bacterial *pmoC* gene(s) in the sample, while “X” indicates no detection.

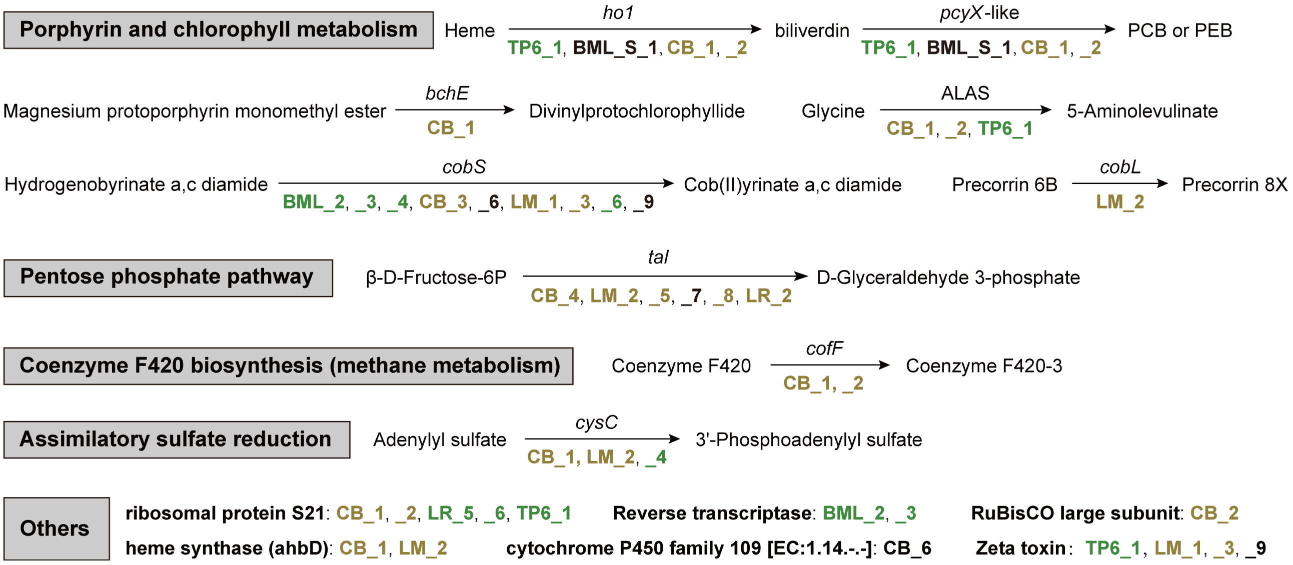


**Supplementary Fig. 22.** Genes of specific metabolic potentials detected in newly reconstructed phage genomes. Abbreviations: *ho1*, heme oxygenase ; *pcyX*-like, phycocyanobilin:ferredoxin oxidoreductase; PCB, phycocyanobilin; PEB, phycoerythrobilin; *bchE*; anaerobic magnesium-protoporphyrin IX monomethyl ester cyclase; ALAS, 5-aminolevulinate synthase; *cobS*; cobaltochelatase CobS; *cobL*, precorrin-6Y C5,15-methyltransferase (decarboxylating); *tal*, transaldolase; cofF, gamma-F420-2:alpha-L-glutamate ligase; cysC, adenylylsulfate kinase.

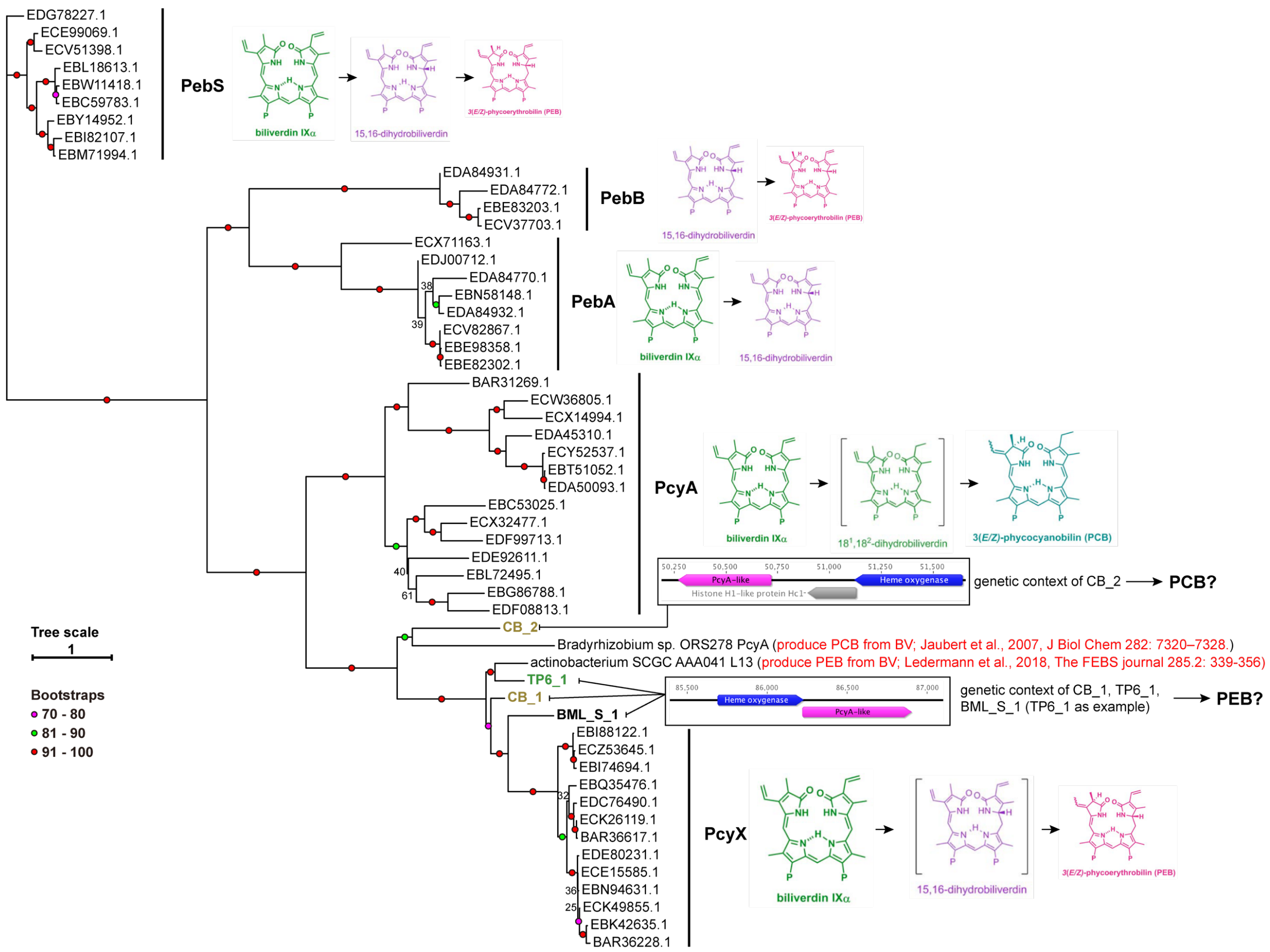


**Supplementary Fig. 23. Phylogenetic analyses of the Pcy-A like proteins encoded by three pmoC-phages and one non-pmoC-phage reported in this study.** The ferredoxin-dependent bilin reductases (FDBRs) reference sequences were retrieved from a previous study [8], along with the information of products from the corresponding proteins. The products of PcyA_Brady and PcyX_actino have been documented and reported [9]. Using this information, the products of similar phage proteins reported in this study were predicted. The genetic context of genes in phages reported in this study is shown in detail. The analyses were performed by first aligning the proteins using Muscle [10] and filtering the alignment by trimAl [11] to remove those columns with ≥ 90% gaps, followed by tree building with IQtree [12] using the “LG+G4” model.

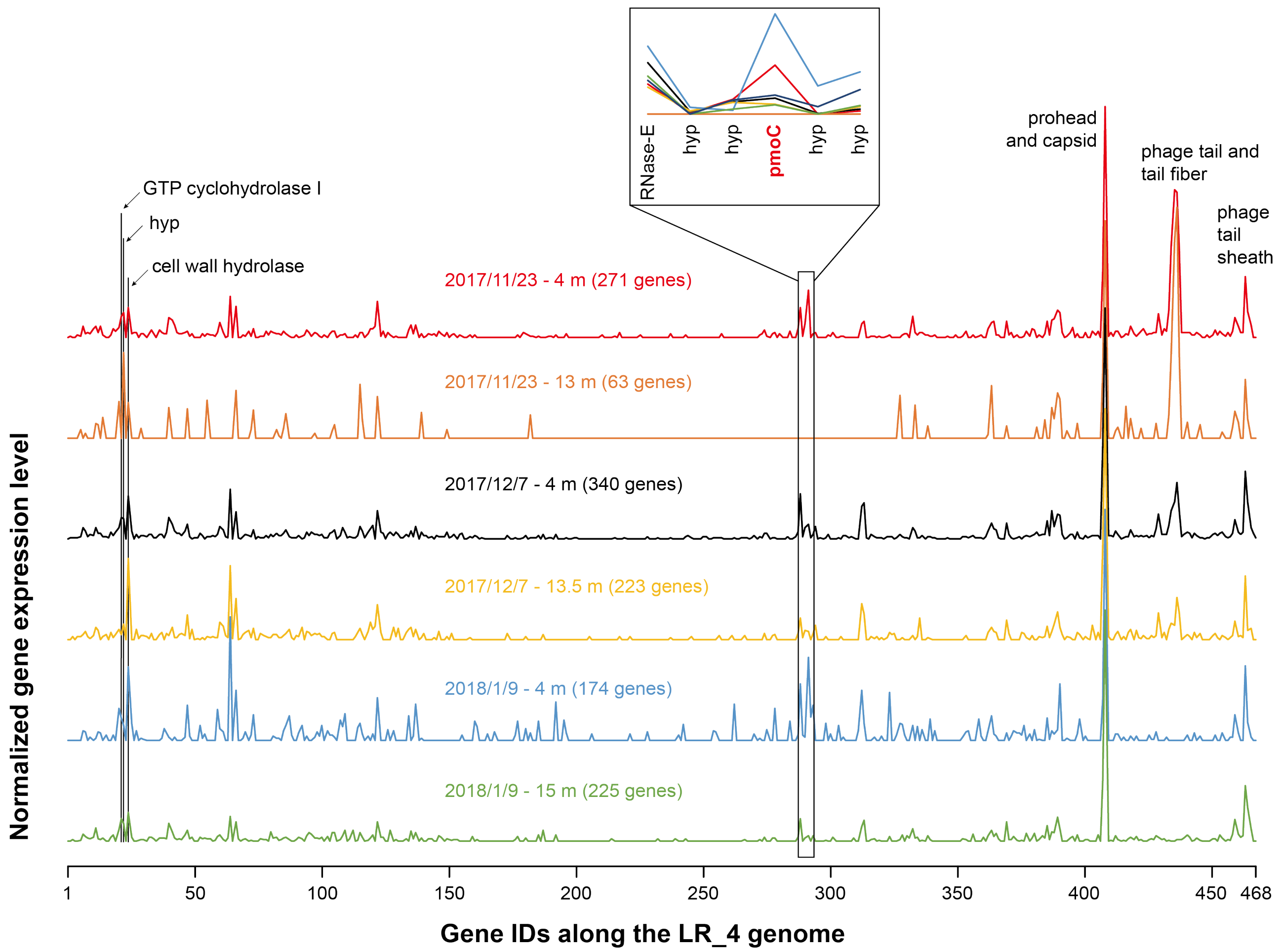


**Supplementary Fig. 24. The expression profiles of genes along the genome of LR_4 from Lake Rotsee.** The sample collection time points are shown for each sample above the gene transcriptional profile, followed by the sampling depth, and the number of genes transcribed in the brackets. Some genes with high transcriptional activities are highlighted with their annotations, the transcriptional levels of six syntenic genes including the *pmoC* genes are shown in the insert.

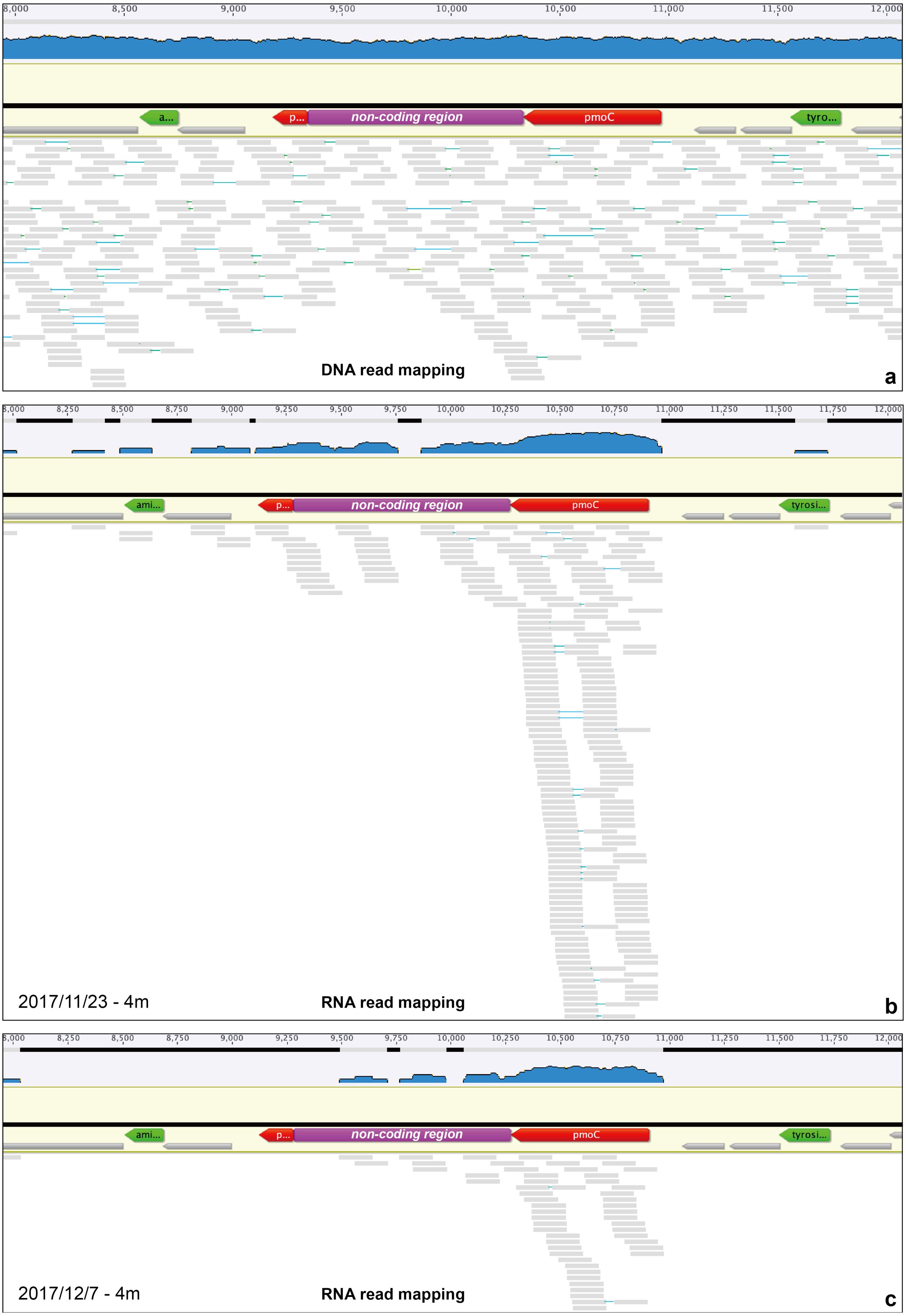


**Supplementary Fig. 25. The assembly and expression of the fragmented *pmoC* gene in LR_6.** (a) DNA read mapping confirms the fragmentation of the *pmoC* gene in LR_6. (b) and (c) The mapping of RNA reads from the “11/23/2017 - 4m” and “2017/12/7 - 4m” samples to the fragmented *pmoC* gene indicates no or low expression level of the smaller fraction, the transcription of the “non-coding region”, and a higher transcriptional level of the larger fraction of the *pmoC* gene.

## Supplementary References


1. Zimmerman, A. E. *et al.* Metabolic and biogeochemical consequences of viral infection in aquatic ecosystems. *Nat. Rev. Microbiol.* **18**, 21–34 (2020).
2. Dammeyer, T., Bagby, S. C., Sullivan, M. B., Chisholm, S. W. & Frankenberg-Dinkel, N. Efficient Phage-Mediated Pigment Biosynthesis in Oceanic Cyanobacteria. *Curr. Biol.* **18**, 442–448 (2008).
3. Ledermann, B. *et al.* Evolution and molecular mechanism of four-electron reducing ferredoxin-dependent bilin reductases from oceanic phages. *FEBS J.* **285**, 339–356 (2018).
4. Thompson, L. R. *et al.* Phage auxiliary metabolic genes and the redirection of cyanobacterial host carbon metabolism. *Proc. Natl. Acad. Sci. U. S. A.* **108**, E757–64 (2011).
5. Kolde, R. Pheatmap: pretty heatmaps. *R package version* **61**, 617 (2012).
6. Mihara, T. *et al.* Linking Virus Genomes with Host Taxonomy. *Viruses* **8**, 66 (2016).
7. Rochman, F. F. *et al.* Benzene and Naphthalene Degrading Bacterial Communities in an Oil Sands Tailings Pond. *Front. Microbiol.* **8**, 1845 (2017).
8. Ledermann, B., Béjà, O. & Frankenberg-Dinkel, N. New biosynthetic pathway for pink pigments from uncultured oceanic viruses. *Environ. Microbiol.* **18**, 4337–4347 (2016).
9. Ledermann, B. *et al.* Evolution and molecular mechanism of four-electron reducing ferredoxin-dependent bilin reductases from oceanic phages. *The FEBS Journal* vol. 285 339–356 (2018).
10. Edgar, R. C. MUSCLE: multiple sequence alignment with high accuracy and high throughput. *Nucleic Acids Res.* **32**, 1792–1797 (2004).
11. Capella-Gutiérrez, S., Silla-Martínez, J. M. & Gabaldón, T. trimAl: a tool for automated alignment trimming in large-scale phylogenetic analyses. *Bioinformatics* **25**, 1972–1973 (2009).
12. Nguyen, L.-T., Schmidt, H. A., von Haeseler, A. & Minh, B. Q. IQ-TREE: a fast and effective stochastic algorithm for estimating maximum-likelihood phylogenies. *Mol. Biol. Evol.* **32**, 268–274 (2015).
