# Supplementary material for: Large freshwater phages with the potential to augment aerobic methane oxidation
Source: Nat Microbiol. 2020 Aug 24;5(12):1504–15. doi: 10.1038/s41564-020-0779-9 (PMC7674155; doi:10.1038/s41564-020-0779-9)
Supplement: Supplementary file 2 — Reporting summary [file 41564_2020_779_MOESM2_ESM.pdf]

nature research

Corresponding author(s): Jillian F Banfield

Last updated by author(s): Jul 7, 2020

Reporting Summary

Nature Research wishes to improve the reproducibility of the work that we publish. This form provides structure for consistency and transparency in reporting. For further information on Nature Research policies, see Authors & Referees and the Editorial Policy Checklist.

Statistics

For all statistical analyses, confirm that the following items are present in the figure legend, table legend, main text, or Methods section.

n/a	Confirmed	
☐	☒	The exact sample size (*n*) for each experimental group/condition, given as a discrete number and unit of measurement
☒	☐	A statement on whether measurements were taken from distinct samples or whether the same sample was measured repeatedly
☐	☒	The statistical test(s) used AND whether they are one- or two-sided<br>*Only common tests should be described solely by name; describe more complex techniques in the Methods section.*
☒	☐	A description of all covariates tested
☒	☐	A description of any assumptions or corrections, such as tests of normality and adjustment for multiple comparisons
☒	☐	A full description of the statistical parameters including central tendency (e.g. means) or other basic estimates (e.g. regression coefficient) AND variation (e.g. standard deviation) or associated estimates of uncertainty (e.g. confidence intervals)
☐	☒	For null hypothesis testing, the test statistic (e.g. *F*, *t*, *r*) with confidence intervals, effect sizes, degrees of freedom and *P* value noted<br>*Give P values as exact values whenever suitable.*
☒	☐	For Bayesian analysis, information on the choice of priors and Markov chain Monte Carlo settings
☒	☐	For hierarchical and complex designs, identification of the appropriate level for tests and full reporting of outcomes
☒	☐	Estimates of effect sizes (e.g. Cohen's *d*, Pearson's *r*), indicating how they were calculated

Our web collection on statistics for biologists contains articles on many of the points above.

Software and code

Policy information about availability of computer code

Data collection	Geneious version 9.0.5 (Licensed, paid version used in this study, free versions available)<br>IDBA_UD version 1.1.1<br>Bowtie2 aligner version 2.3.5.1
Data analysis	Prodigal V2.6.3<br>usearch v10.0.240_i86linux64, 1057Gb RAM, 80 cores<br>tRNAscan-SE 2.0<br>MUSCLE v3.8.31<br>BLASTp version 2.10.0+<br>BLASTn version 2.10.0+<br>hmmsearch (built in HMMER 3.3)<br>ra2.py<br>minced V0.4.2<br>MMseqs2<br>HHpred version 3.0.3<br>IQtree version 1.6.12<br>BBTools version 37.50<br>sickle version 1.33

For manuscripts utilizing custom algorithms or software that are central to the research but not yet described in published literature, software must be made available to editors/reviewers. We strongly encourage code deposition in a community repository (e.g. GitHub). See the Nature Research guidelines for submitting code & software for further information.

Data

Policy information about availability of data

All manuscripts must include a data availability statement. This statement should provide the following information, where applicable:

- Accession codes, unique identifiers, or web links for publicly available datasets
- A list of figures that have associated raw data
- A description of any restrictions on data availability

The genomes of pmoC-phages and their relatives reported in this study have been deposited at NCBI under PRJNA645206, and are also available at Figshare (https://figshare.com/projects/pmoC-phages_in_freshwater_ecosystems/76623). The read archive and other accession information is provided in Supplementary Table 8. The pmoACB and Cas proteins HMM datasets are available at http://tigrfams.jcvi.org/cgi-bin/Listing.cgi. The 16S rRNA gene HMM database is available at https://github.com/christophertbrown/bioscripts/tree/master/databases.

Field-specific reporting

Please select the one below that is the best fit for your research. If you are not sure, read the appropriate sections before making your selection.

☒ Life sciences ☐ Behavioural & social sciences ☐ Ecological, evolutionary & environmental sciences

For a reference copy of the document with all sections, see nature.com/documents/nr-reporting-summary-flat.pdf

Life sciences study design

All studies must disclose on these points even when the disclosure is negative.

Sample size	Base Mine Lake (BML): N = 28 samples, each sample was collected and sequenced individually.<br>BML source (BML_S): N = 1 sample, the sample was collected and sequenced independently.<br>Lake Mendota (LM): N = 91 samples, the samples were from Lake Mendota in Wisconsin, USA in 2008-2012.<br>Crystal Bog (CB): N = 79 samples, the samples were from Crystal Bog in Wisconsin, USA in 2007-2009.<br>Lake Rotsee (LR): N = 6 samples, all with both metagenomic and metatranscriptomic datasets from Lake Rotsee of Switzerland (2017 and 2018).<br><br>We analyzed all the datasets that were collected in this study (i.e., BML and BML_S) and those reported in previous studies (i.e., LM, CB, LR). We did not select samples from them. As we are searching the existence of pmoC-phages in the corresponding samples, the sample sizes are sufficient for presence and absense analyses.
Data exclusions	None
Replication	Sample collection was not replicated.
Randomization	Randomization is not applicable because there were no experimental groups designated in this study.
Blinding	Blinding was not performed because it was not applicable to this study. This study was a survey of various populations, and was not dependent on the presence / absence of certain characteristics.

Reporting for specific materials, systems and methods

We require information from authors about some types of materials, experimental systems and methods used in many studies. Here, indicate whether each material, system or method listed is relevant to your study. If you are not sure if a list item applies to your research, read the appropriate section before selecting a response.

Materials & experimental systems

n/a	Involved in the study	
☒	☐	Antibodies
☒	☐	Eukaryotic cell lines
☒	☐	Palaeontology
☒	☐	Animals and other organisms
☒	☐	Human research participants
☒	☐	Clinical data

Methods

n/a	Involved in the study	
☒	☐	ChIP-seq
☒	☐	Flow cytometry
☒	☐	MRI-based neuroimaging
